# Defective CFTR leads to aberrant β-catenin activation and kidney fibrosis

**DOI:** 10.1038/s41598-017-05435-5

**Published:** 2017-07-12

**Authors:** Jie Ting Zhang, Yan Wang, Jun Jiang Chen, Xiao Hu Zhang, Jian Da Dong, Lai Ling Tsang, Xiao Ru Huang, Zhiming Cai, Hui Yao Lan, Xiao Hua Jiang, Hsiao Chang Chan

**Affiliations:** 1 Epithelial Cell Biology Research Center, Key Laboratory for Regenerative Medicine of Ministry of Education of China, School of Biomedical Sciences, Hong Kong, People’s Republic of China; 2 Li Ka Shing Institute of Health Sciences, Faculty of Medicine, The Chinese University of Hong Kong, Hong Kong, People’s Republic of China; 3 School of Biomedical Sciences Core Laboratory, Shenzhen Research Institute, The Chinese University of Hong Kong, Shenzhen, 518057 People’s Republic of China; 4 Shenzhen Second People’s Hospital, The First Affiliated Hospital of Shenzhen University, Shenzhen, People’s Republic of China; 5 Key Laboratory of Reproduction and Genetic of Ningxia Hui Autonomous Region, Key Laboratory of Fertility Preservation and Maintenance of Ningxia Medical University and Ministry of Education of China, Yinchuan, People’s Republic of China

## Abstract

Cystic fibrosis transmembrane conductance regulator (CFTR), known as a cAMP-activated Cl^−^ channel, is widely expressed at the apical membrane of epithelial cells in a wide variety of tissues. Of note, despite the abundant expression of CFTR in mammalian kidney, the role of CFTR in kidney disease development is unclear. Here, we report that CFTR expression is downregulated in the UUO (unilateral ureteral obstruction)-induced kidney fibrosis mouse model and human fibrotic kidneys. Dysfunction or downregulation of CFTR in renal epithelial cells leads to alteration of genes involved in Epithelial-Mesenchymal Transition (EMT) and kidney fibrosis. In addition, dysregulation of CFTR activates canonical Wnt/β-catenin signaling pathways, whereas the β-catenin inhibitor reverses the effects of CFTR downregulation on EMT marker. More interestingly, CFTR interacts with Dishevelled 2 (Dvl2), a key component of Wnt signaling, thereby suppressing the activation of β-catenin. Compared to wild type, *delta*F508 mice with UUO treatment exhibit significantly higher β-catenin activity with aggregated kidney fibrogenesis, which is reduced by forced overexpression of CFTR. Taken together, our study reveals a novel mechanism by which CFTR regulates Wnt/β-catenin signaling pertinent to progression of kidney fibrosis and indicates a potential treatment target.

## Introduction

Kidney fibrosis is a common histological manifestation of functional decline in most cases of end-stage kidney diseases. Fibrosis is a reactive process that develops in response to excessive epithelial injury and inflammation, leading to myofibroblast activation and an accumulation of extracellular matrix (ECM)^1–3^. While the cellular origin of myofibroblasts remains to be a subject of intensive debate, numerous animal and clinical studies have pointed to epithelial-to-mesenchymal-transition (EMT) from the tubular epithelial cells as one of the major contributors leading to the generation of myofibroblasts and overproduction of interstitial ECM^4–9^. A multitude of biochemical signaling pathways has been proposed to be associated with the development of kidney fibrosis^10–12^. While Wnt/β-catenin signaling is functionally important in normal kidney development^13, 14^, aberrant activation of Wnt/β-catenin signaling and subsequent downstream targets, such as matrix metalloproteinases (MMPs), fibronectin and snail, has been observed in the progression of tubulointerstitial kidney fibrosis in unilateral ureteral obstruction (UUO) model^15–17^ and different types of kidney diseases^18–21^. Of interest, β-catenin is predominantly up-regulated in renal tubular epithelium of the fibrotic kidneys, suggesting that tubular cells are the major targets of canonical Wnt signaling. In addition, activation of β-catenin in tubular epithelial cells induces EMT, as well as the expression of several fibrosis-related genes *in vitro* ^22–24^. These results suggest that activation of tubular β-catenin probably plays a critical role in the pathogenesis and progression of kidney fibrosis^16^. However, it remains unclear how the Wnt/β-catenin signaling pathway is over-activated in fibrotic kidney, and how this signal initiated from the tubular epithelial cells is converted into pathological changes in the tubulo-interstitial space leading to excessive deposition of ECM and fibrogenesis.

Cystic fibrosis transmembrane conductance regulator (CFTR), known as a cAMP-activated Cl^−^ channel, is widely expressed at the apical membrane of epithelial cells in a wide variety of tissues^25, 26^. Dysfunction of CFTR causes cystic fibrosis (CF), the most common lethal autosomal recessive disease in Caucasians^27^. Of note, despite the abundant expression of CFTR in mammalian kidney which is localized at the apical surface of proximal tubules and distal tubules^28^, no overwhelming primary renal disease has been associated with CF. Nevertheless, as CF patients age and have an increased exposure to potentially nephrotoxic agents, such as antibiotics and high glucose, development of chronic renal disease has been commonly recognized^29, 30^. Given that CF-related nephropathies are frequently compounded by increasing age, pulmonary infection, malnutrition, liver dysfunction and insulin insufficiency^31^, chronic kidney damage has been considered as secondary manifestation due to the abnormalities in salt transport, infection, and development of cystic fibrosis related diabetes (CFRD)^29, 32^. One study involving 12 000 patients followed up for a median of 4 years reported an overall annual prevalence of stage 3 chronic kidney disease of 2.3%^33^. In addition, permanent microalbuminuria occurs in more than 6% of patients with cystic fibrosis^30^. Despite all these link, the exact role of CFTR in the pathogenesis of chronic kidney diseases has never been investigated.

Interestingly, a recent comprehensive study on human lung epithelial cells has found that the CFTR interactome includes several hundred proteins, with β-catenin listing as a potential interacting protein^34^. In addition, both our group and other groups have reported that CFTR interacts with β-catenin in intestinal epithelial cells, dysregulation of which leads to aggregated inflammation and development of cancer^35, 36^. On the other hand, TGF-β and hypoxia inducing factor α (HIFα), key inducers of kidney fibrosis, have been demonstrated to suppress the expression and/or localization of CFTR in the context of epithelial cells^37–39^. Based on these observations, we hypothesized that dysregulation of CFTR might lead to aberrant activation of Wnt/β-catenin pathway and development of kidney fibrosis. We undertook the present study to investigate this possibility using kidney epithelial cell lines, UUO mouse model and CFTR mutant mice in conjunction with genetic manipulation *in vitro* and *in vivo*.

## Materials and Methods

### Cell lines and treatment

Renal distal tubular Madin–Darby canine kidney (MDCK) (ATCC, CRL-2936) cells were cultured in α-MEM medium (GIBCO, NY, USA), supplemented with 10% Fetal Bovine Serum (FBS). Human proximal tubule (HK-2) cells were cultured in DMEM/F12 medium (GIBCO, NY, USA), supplemented with 10% FBS. Cells were maintained in 5% CO_2_ at 37 °C, and seeded into 6 well plates at 5 × 10^4^/well and incubated in growth medium supplemented with 2% FBS during treatment. CFTR_inh_-172 (Sigma, MO, USA) was used at the final concentration of 10 µM, whereas GlyH101 (Santa Cruz, California, USA) was used at 2.5 μM, 5 μM and 10 μM for 2 or 3 days as indicated. DMSO (0.1%) at the same volume was used as vehicle control. The effect of hypoxia was achieved by incubating cells in 1% O_2_–5% CO_2_ - balance N_2_ chamber for 1 and 2 days before further assay. Cells incubated under normoxia condition were used as control.

### Plasmids and transient transfection

To knockdown CFTR expression in MDCK cells, miRNAs duplex specific to canis familiaris CFTR was synthesized by Life Technologies (Carlsbad, CA). The siRNA sequence is as follows: 5′- ACT TGT TAC TCC TTT CCA A -3′. MiRNA against Lac-z was used as the control sequence. pcDNA^TM^ 6.2-GW/EmGFP encoding a pre-miRNA sequence was constructed using BLOCK-iT^TM^ Pol II miR RNAi Expression Vector kit (Life technologies). MDCK cells were seeded on 35 mm culture dishes and 3 μg of vectors were transiently transfected with 6 μl Lipofectamine 2000 (Invitrogen) following the manufacturer’s instruction. The GFP signals were used to evaluate the transfection efficiency. 48 hours after transfection, cell lysates were collected for protein assay to confirm the transfection efficiency.

The ribozymes were generated and used for CFTR knockdown in HK-2 cells as described in our previous study^40^. For knockdown experiments, 3 μg DNA was transfected into HK-2 cells with 6 μl Lipofectamine 2000 (Invitrogen) and selected by 8 μg/ml Blasticidin S for 2–3 weeks. The stable transfectants were maintained in medium containing 4 μg/ml Blasticidin S.

### Real time-PCR

Total RNA was isolated and reversely transcribed using standard protocols. For quantitative PCR, assays were performed in triplicate on an Applied Biosystems 7500 Fast Real-Time PCR System. The primers were listed in Supplementary Tables 1 and 2.

### Co-immunoprecipitation

Protein was extracted using lysis buffer (20 mM Tris HCl pH 8, 137 mM, NaCl10% glycerol, 1% Nonidet P-40 (NP-40), 2 mM EDTA and protease inhibitor). Cell lysates was incubated with primary antibody: anti-CFTR (Millipore MAB3482, clone MM13-4; US) or anti-Dvl2 (Cell signaling technology CST 3224 S, clone 30D2; US). After incubation, the complexes were precipitated using the protein A/G plus-agarose immunoprecipitation reagent, washed and eluted for Western blot assay.

### MTS assay

3000 cells/well in 200 μL growth medium were seeded into 96-well plate. Following the appropriate incubation period, medium was removed and 100 μL full medium with 20 μL MTS (3-(4, 5-dimethylthiazol-2-yl)-5-(3carboxymethoxyohenyl)-2-(4-sulfop henyl)-2H-tetrazolium, Promega) reagent was added. The experimental plate was further incubated for 2 hours to allow the bio-reduction of metabolically active cells. The intensity of soluble product was measured by spectrophotometer at 490 nm. Three replicated wells were measured for each experimental group.

### Western blot analysis

Cell or tissue protein were extracted using lysis buffer [RIPA: 50 mM Tris, 150 mM NaCl, 1% Triton X-100, 0.1% SDS and 1% nadeoxycholate (pH 7.4)] supplemented with protease inhibitors including phenylmethylsulfonyl fluoride and pimix. The samples were incubated with RIPA at room temperature for 30 minutes for CFTR detection, or boiled at 100 °C for 3 minutes for other protein detection. Total lysates of cells (20 µg per lane) or tissues (40 µg per lane) were then subjected to SDS–polyacrylamide gel electrophoresis and were transferred onto nitrocellulose membranes (Hybond^TM^-P; Amersham Biosciences, Piscataway, NJ). The transferred membrane was blocked with TBS containing 0.2% Tween-20 (TBST) and 5% non-fat dry milk, and incubated with primary antibodies as follow: anti-CFTR (alomone labs ACL-006; US); anti-HIF-1α (Novus Biologicals NB100-449; US); EMT markers including anti-E-cadherin (Santa cruz sc-7870, clone H108; US), anti-Occludin (invitrogen 711500; US), anti-ZO-1 (invitrogen 617300, US), anti-Vimentin (Santa cruz sc-5565, clone H84; US), anti-N-cadherin (Invitrogen 333900; US) and anti-α-SMA (AbCAM ab5694; US), anti-Fibronectin (Santa Cruz sc-56391, clone SPM246; US), anti-Collagen1 (SouthernBiotech #1310-01; kindly from Prof Hui Yao LAN); Wnt/β-catenin signaling including Wnt signaling antibody sampler kit (Cell signaling technology 2915; US), Wnt/β-catenin activated targets antibody sampler kit (Cell signaling technology 8655; US), anti-β-catenin (Cell signaling technology 9562; US) at 4 °C overnight. Antibody against GAPDH (santa cruz sc-47724, clone 0411; US), β-tubulin (Santa Cruz sc-9104, clone 235; US) or Histone H1(Santa Cruz sc-8030, clone AE-4; US) were used for protein loading controls. The membrane was subsequently washed with TBST and incubated for 1 h with peroxidase-conjugated secondary antibodies. The membrane was washed three times with TBST and then detected by enhanced chemiluminescence (Amersham, Piscataway, NJ, USA).

### Trans- epithelial electric resistance (TER)

MDCK cells were seeded into the 0.4 mm pore size transwell (upper chamber) and allowed to reach full confluence, after which fresh medium was replaced for further experiments. TER of epithelial monolayer was measured using EVOM electrovoltohmeter (WPI, Sarasota, FL) every two days following the standard procedure. Three replicated wells were used for measurement at each time point.

### Wound-healing migration assay

Cells were seeded at 5 × 10^5^ cells/well in 6-well plate one day before creating a wound across the cell monolayer with a plastic tip. Closure of the wound via the migration of cells was tracked and recorded using a Time Lapse Imaging System (CarlZwiss). Five 100× views from each well were documented and the results were expressed as reduced areas of wound. The experimental procedure was repeated three independent times.

### Luciferase Activity Assay

The plasmids pcDNA3.1 and pcDNA3.1-CFTR were transiently co-transfected to HK-2 with TOPflash reporter gene (0.05 ug/ml) (Millipore, USA) by Lipofectamine 2000 (Invitrogen). Renilla-Luc (0.005 ug/ml) was included in all transfection assays as an internal control. Firefly and renilla luciferase activity was assessed with respective assay kit (Promega). The relative luciferase activity was defined as the ratio of readout for firefly luciferase to that for renilla luciferase with that of control group set as 1.0. Triplicate wells were used for measurement at each group. The experimental procedure was repeated three independent times.

### Animals and Unilateral ureteric obstruction (UUO) model

All mice were provided by the Laboratory Animal Service Center of the Chinese University of Hong Kong. All animal experiments were conducted in accordance with the University Laboratory Animals Service Center’s guidelines on animal experimentation with approval from the Animal Ethnics Committee of the University. CFTR *delta*F508 mouse model (The Jackson Laboratory, B6.129S6-*Cftr* ^tm1Kth^) generated on C57BL/6 background^41^ was used in the study. KAPA Mouse Genotyping Kits (KAPA Biosystem) are used for genotyping followed standard protocol. Briefly, DNA was extracted from 3–5 mm mouse tail by the KAPA Express Extract (a thermostable protease and buffer system). The supernatant was then diluted 10-fold prior to use as template in PCR reaction (Figure S5b, Table S2). Homozygous mutant mice (*delta*F508) were reduced in size compared to that of normal wild type mice (wt) and heterozygous (*delta*F508 hetero). Mutant mice showed an increased mortality within the first month after birth (~60%), due to bowel obstructions, bowel strictures and peritonitis. Regardless of the survival rate, the animal lungs, pancreas, gall bladder, male reproductive tract, lacrimal gland and submandibular glands from *delta*F508 mice appear normal.

Unilateral ureteric obstruction (UUO) model was established in female wild type and CFTR *delta*F508 heterozygous (hetero), *delta*F508 homozygous (homo); male wild type and *smad3* knockout mice (provided by Prof. Hui Yao Lan, the Chinese University of Hong Kong) at 8-week-old. Six mice were used in each group. UUO model was established by left ureter ligation as previously described^42^. Mice were euthanized at Day 3 or 7, after the ligation. Kidney tissue samples were collected for histology, immunohistochemistry and real-time PCR.

The male C57 mice at 8-week-old were provided for the Ultrasound-mediated gene transfer within the kidney during fibrosis. The plasmid peGFP-C3 and peGFP-CFTR for injection were prepared using the EndoFree plasmid kit (Qiagen Inc., Valencia, CA, USA) according to the manufacturer’s instructions. Before injection, 200 μg of peGFP-C3 or peGFP-CFTR plasmid were mixed with sulphur hexafluoride microbubbles (Bracco Imaging B.V., Geneva, Switzerland) at a ratio of 1:1 (v/v). The mixed solution (200 μl) was injected into tail vein. Ultrasound was performed immediately after injection with a continuous-wave output of 1 MHZ for 5 min on the back of left kidney location. Unilateral ureteric obstruction (UUO) model was established following the ultrasound treatment. A group of 9 or 10 mice were euthanized at Day 7, after the ligation. Kidney tissue samples were collected for histology, immunohistochemistry, western blot and real-time PCR.

### HE and Masson’s trichrome stain

Changes in renal morphology were examined in paraformaldehyde fixed, paraffin-embedded tissue sections (5 μm) stained with haematoxylin and eosin or Modified Masson’s Trichrome Stain Kit (ScyTek Laboratories). Briefly, deparaffinized sections were incubated in Bouin’s Fluid overnight. After wash by tap water, slides were stained sequentially by working Weigert’s Iron Hematoxylin for 5 mins, Biebrich Scarlet/Acid Fuchsin solution for 30 s, and Aniline Blue Solution for 45 mins.

### Immunofluorescent and immunohistochemistry staining

MDCK or HK-2 cells were seeded into 24-well plate with cover slips for immunofluorescent assay. After treatment, cells were fixed with 4% paraformaldehyde and blocked with 1% bovine serum albumin, and incubated overnight with primary antibodies at a dilution of 1:100 in 1% BSA against ZO-1, Vimentin, N-cadherin, CFTR (Santa Cruz sc-8909, clone N-20; US) or Dvl2 at 4 °C. For paraffin embedded tissues, steps for rehydration and microwave-based antigen retrieval^43^ were performed before incubation with primary antibody against CFTR or HIF-1α. Then secondary Alexa fluor 568 goat anti-mouse IgG, fluor 488 goat anti-rabbit IgG, fluor 488 donkey anti-goat IgG or fluor 568 donkey anti-rabbit IgG at a dilution of 1:500 was loaded on cells or tissues for 1 hour. Samples were all co-stained with Hoechst for nuclei detection. Eventually, the slides were mounted with ProLong Gold Antifade Reagent (Invitrogen) and visualized with fluorescence microscope (Nikon Intersilight C-HGF1) using 4×, 10×, 20× and 40× objectives.

Immunohistochemistry was also performed in paraffin sections following the similar antigen retrieval procedure^43^. The primary antibodies used in this study include Collagen I and F4/80(AbD Serotec MCA497G, US). All slides were further stained with haematoxylin for the nuclei. 15 to 20 views were taken randomly by the Nikon 20× and 40× objectives of every slide for the quantitative analysis. Area of Collagen I staining was quantitated by the image analysis software (Meta Imaging series 7.5) on 200× fields. Briefly, the area of the positive staining pattern in tubulointerstitium was identified. Then, the percentage of positive area in the examined view was measured. The number of macrophages with the positive staining of F4/80 was counted in 400× fields, and the numbers were shown as cells/mm^2^. All analysis data was expressed as the Mean ± S.E.M. from three animals at indicated time point for each group.

### Statistical analysis

All morphometric and staining data were collected blindly. Statistical significance for comparison between two measurements was determined using the unpaired Two-tailed Student t-test. One-way or Two-way ANOVA was used for evaluation of the significance among three or more measurements. All statistical analysis was completed by Prism 5 (GraphPad, Inc., San Diego, CA, USA). Values of p < 0.05 were regarded as statistically significant, and 0.01 < p < 0.05 was labeled as ‘*’, 0.001 < p < 0.01 was labeled as ‘**’ and p < 0.001 was labeled as ‘***’. All statistical data were shown as Mean ± S.E.M.

## Results

### Downregulation of CFTR in murine and human fibrotic kidney

**Figure 1:**
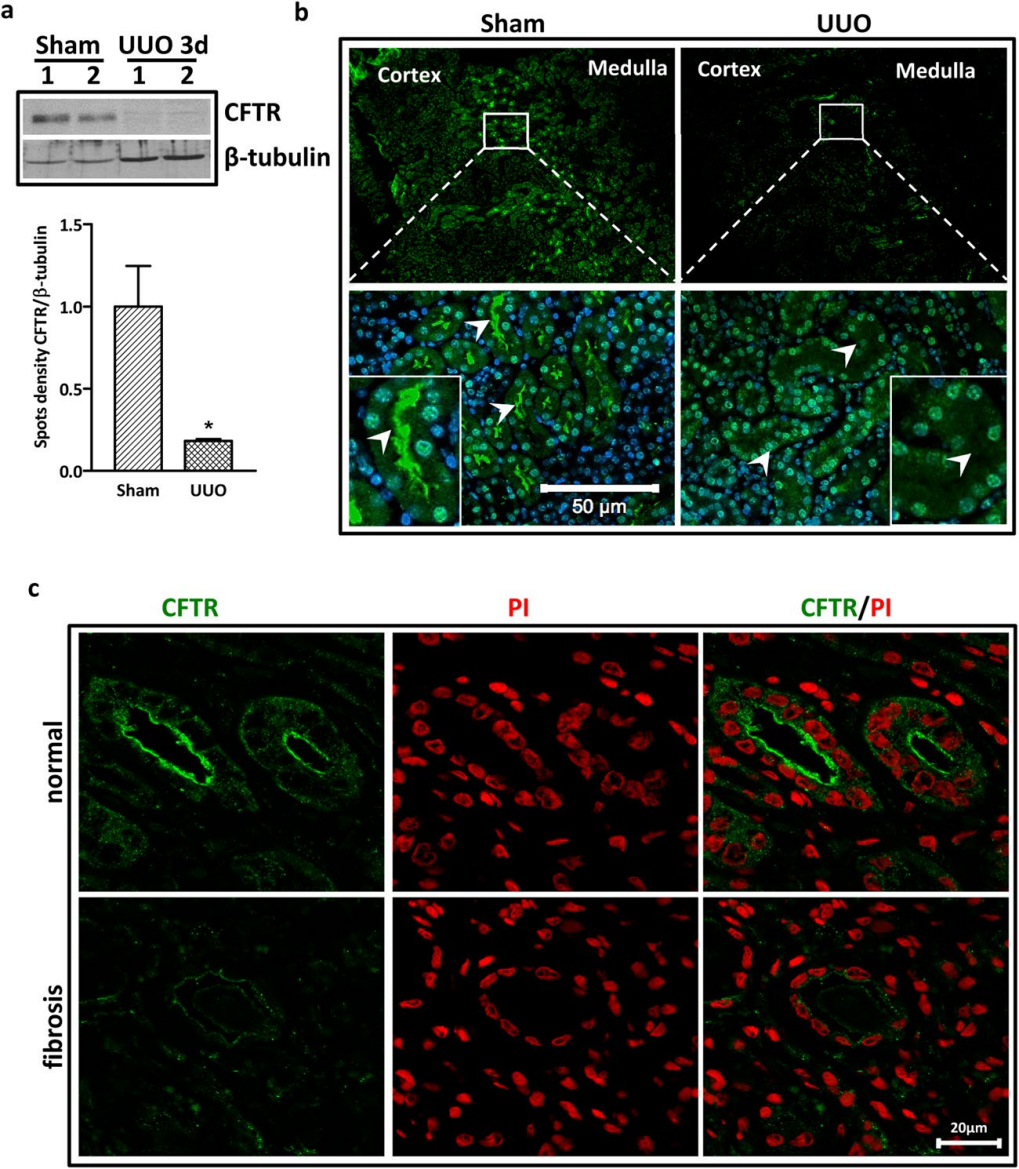
Downregulation of CFTR in UUO mouse model and human fibrotic kidneys. (**a**) Representative western blot showing significantly reduced CFTR expression in UUO kidneys (n = 4) compared to wild type kidneys (n = 5) after 3 days of ligation, quantification is shown in the lower panel, *p < 0.05; (Full-length blot is shown in Supplementary Figure S7a.) (**b**) Immunofluorescent staining showing significantly downregulated CFTR protein in the UUO kidneys compared to the wild type kidneys. Note that in normal kidney, CFTR is expressed in the apical membrane of proximal tubule cells at the corticomedullary junction (white arrow), whereas in UUO kidney, CFTR expression is dramatically downregulated and diffusely localized in the cytoplasm (white arrow), scale bar = 50 µm; (**c**) Immunofluorescent staining shows that CFTR protein is expressed at cell-cell adhesion and cytoplasm in tubular epithelium in normal human kidney. However, the expression of CFTR is significantly decreased in fibrotic kidney tissue (n = 2), scale bar = 20 µm. PI: Propidium Iodide.

To test this hypothesis, we first examined whether CFTR expression was altered during the development of renal interstitial fibrosis by using unilateral ureteral obstruction (UUO) mouse model. Our western blot result showed that the expression of CFTR was dramatically decreased in fibrotic kidneys compared to normal kidneys (Fig. 1a). Consistently, while CFTR was extensively expressed on the apical surface of tubular lumen in the cortex and medulla in control kidney, UUO treatment drastically suppressed the expression level of CFTR (Fig. 1b). The expression of CFTR was further determined in normal and fibrotic human kidneys. In line with the mouse data, CFTR was expressed strongly at the apical membrane of tubular lumen in normal human kidney whereas dramatically downregulated in fibrotic kidney (Figs 1c and S1a). These data suggest that downregulation of CFTR is associated with the development of kidney fibrosis.

### Hypoxia-downregulated CFTR induces upregulation of EMT markers independent of TGF-β

**Figure 2:**
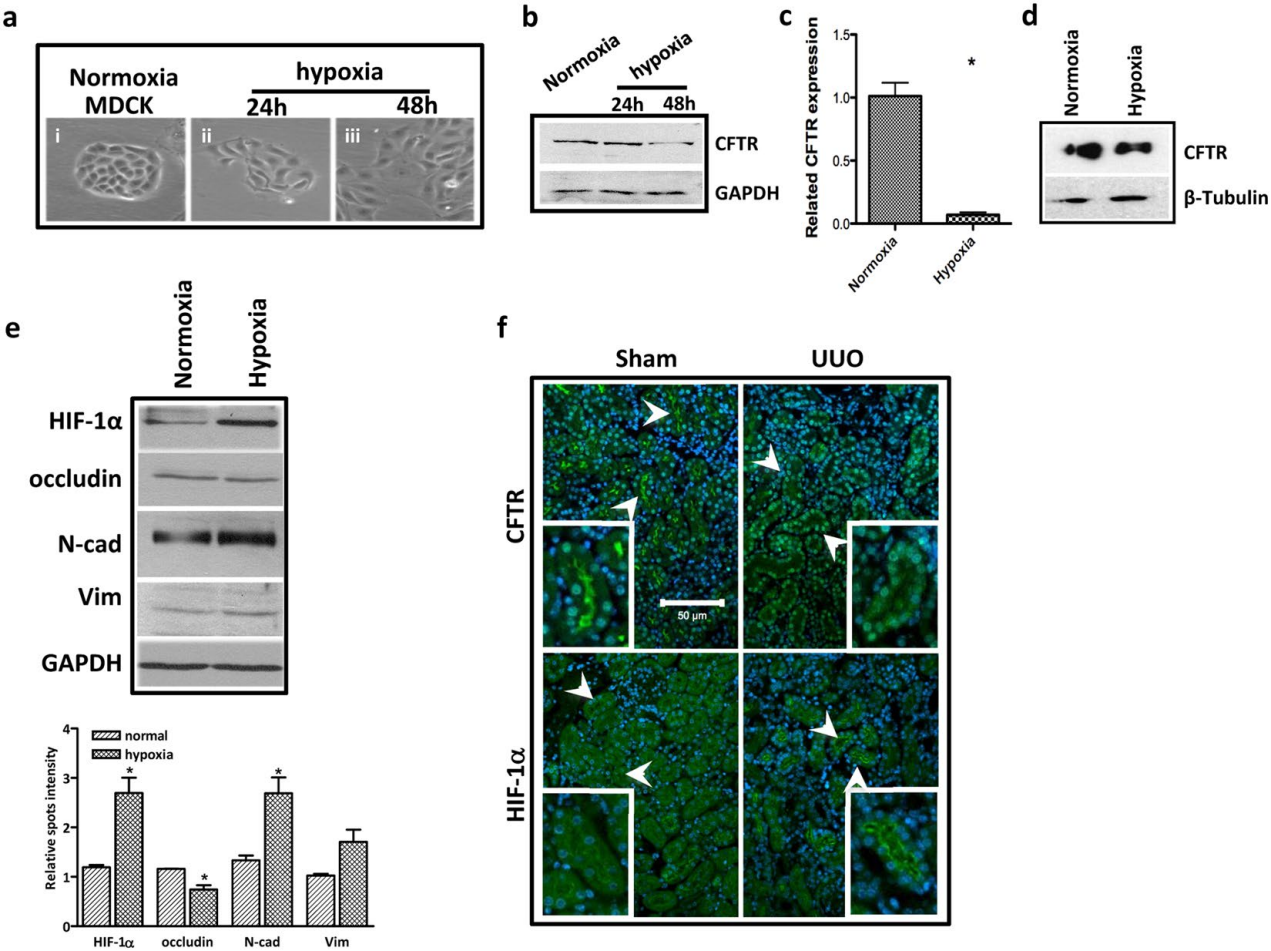
Hypoxia downregulates CFTR expression. (**a**) Phase-contrast photographs of MDCK cells treated with normoxia and hypoxia conditions; (**b**) Western blot showing downregulation of CFTR in the MDCK cells by hypoxia (Full-length blot is shown in Supplementary Figure S7b.); (**c**) Real time-PCR assay showing decreased mRNA expression of CFTR induced by hypoxia in HK-2 cells,*p < 0.05; (**d**) Western blot showing decreased expression of CFTR induced by hypoxia in HK-2 cells (Full-length blot is shown in Supplementary Figure S7c.); (**e**) Western blot showing the expression changes of HIF-1α and EMT markers induced by hypoxia in MDCK cells, quantification analysis is shown in the lower panel, *p < 0.05; (Full-length blot is shown in Supplementary Figure S7d.) (**f**) Immunofluorescent staining showing dramatically increased HIF-1α and reduced CFTR protein levels in tubular epithelial cells at inner cortices in UUO kidney (arrow).

Next, we asked whether CFTR could be downregulated by factors known to induce EMT and kidney fibrosis. Since hypoxia is a major mediator of EMT during kidney fibrosis^44^, we investigated the influence of this pathophysiological important factor on the expression of CFTR in Madin-Darby canine kidney cell line MDCK and human tubular epithelial cell line HK-2, which are widely used as *in vitro* models to study EMT and renal dysfunction^45^. Our result showed that when subjected to hypoxia (1% O_2_), MDCK cells took on an elongated, fibroblastic morphology, which is reminiscent of a mesenchymal transition phenotype (Fig. 2a). Of note, the expression of CFTR was significantly downregulated in MDCK cells 48 hours after hypoxia treatment (Fig. 2b). The downregulation of CFTR was also observed in HK-2 cell by both real-time PCR and western blot analyses (Fig. 2c and d). In addition, the expression of hypoxia-inducible factor-1α (HIF-1α), a well-known critical mediator of hypoxia that has been identified as a negative regulator of CFTR^39^, was significantly increased along with alteration of EMT markers in the MDCK cells (Fig. 2e). To further correlate CFTR expression with hypoxia, we examined the expression of CFTR and HIF-1α in our UUO-treated mouse kidney tissues. Our results showed that the expression of HIF-1α was dramatically increased in the tubulointerstitial space at inner cortices 3 days after ligation in UUO mice, at which downregulation of CFTR was most significant (Fig. 2f). Taken together, downregulation of CFTR expression in both mouse and human kidney fibrotic tissues and downregulation of CFTR by hypoxia, along with upregulation of EMT markers suggest possible involvement of CFTR in EMT and progression of kidney fibrosis.

TGF-β has been considered as another major mediator of EMT in injured kidney^41, 46^. We further tested whether CFTR was involved in the TGF-β-induced EMT in renal epithelial cells. As shown in Supplementary Figure 1b, 5 ng/ml TGF-β_1_ readily induced EMT in MDCK cells as demonstrated by the significant upregulation of N-cadherin and Vimentin whereas downregulation of Occludin after 48–72 hours. However, we noticed that TGF-β_1_ did not have any effect on the expression level of CFTR. To further explore the possible correlation of TGF-β_1_ signaling with CFTR, we took advantage of Smad3 knockout (Smad3 KO) mouse model. Intriguingly, we found that the UUO treatment-induced effect on HIF-1α and CFTR was similar in both Smad3 KO mice and WT mice (Figure S1c), excluding the involvement of TGF- β/Smads pathway. We also determined the effect of CFTR inhibitors on TGF-β signaling and found that CFTR inhibitors did not affect the expression of phosphor-Smad2 (Figure S1d). Taken together, these data imply that CFTR downregulation during renal fibrosis is not related to TGF-β signaling.

### Inhibition/knockdown of CFTR triggers EMT process

**Figure 3:**
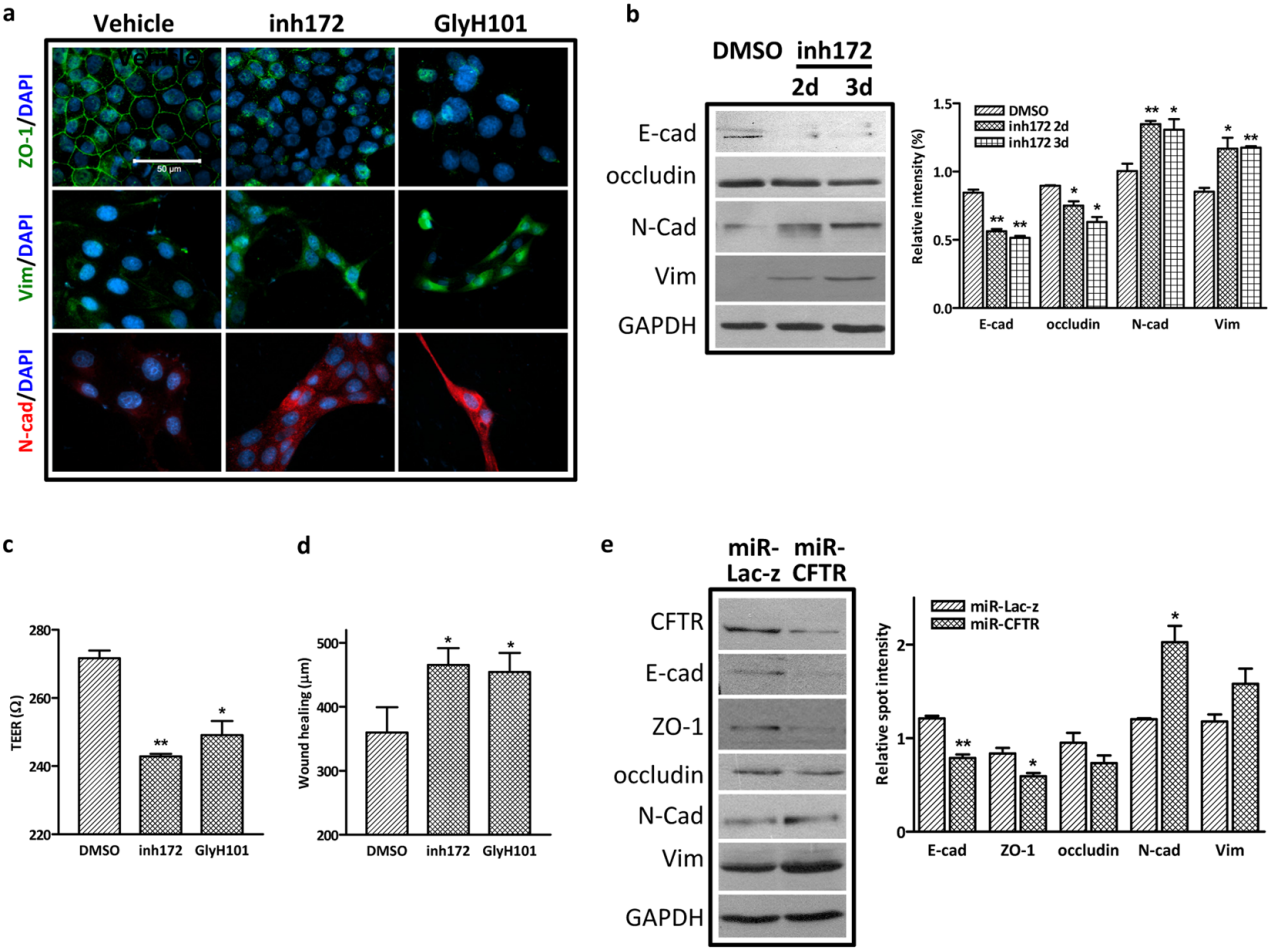
Suppression of CFTR induces EMT in MDCK cells. (**a**) Immunofluorescence staining showing decreased expression of ZO-1 and increased expression of N-cadherin and Vimentin in inh172 (10 µM for 2 or 3 days)- or GlyH101- (2.5 µM, 5 µM and 10 µM for 1 day) treated MDCK cells. Scale bar = 50 µm; (**b**) Western blot analysis showing increased expression of N-cadherin and Vimentin, and decreased expression of E-cadherin and Occludin in 10 µM inh172-treated MDCK cells. (Full-length blot is shown in Supplementary Figure S7e.) Quantification analysis is shown in the right panel, *p < 0.05, **p < 0.01; (**c**) TER (Transepithelial resistance) is significantly lower in CFTR inhibitor-treated MDCK cells. *p < 0.05, **p < 0.01, n = 3; (**d**) Wound healing assay showing enhanced cell migration at 24 hours with CFTR inhibitors treatment. At least 4 random fields per assay were counted and triplicate wells were set up for each sample. *p < 0.05; (**e**) Western blot showing decreased expression of ZO-1, E-cadherin and increased expression of N-cadherin and vimentin in CFTR knockdown cells. (Full-length blot is shown in Supplementary Figure S7f.) Quantification is shown in the right panel, *p < 0.05; **p < 0.01.

We further determined whether CFTR dysfunction or downregulation is a key event leading to EMT and kidney fibrosis. Firstly, we treated MDCK cells with two CFTR inhibitors, CFTR_inh_-172 (inh172) or GlyH101, and found marked morphological changes indicative of EMT in MDCK cells. CFTR inhibitor-treated cells had a greater predilection to break away as single cells and lose cell-cell contacts (Fig. 3a and Figure S2a). In addition, inhibition of CFTR significantly downregulated epithelial markers and upregulated mesenchymal markers in a time- and dose-dependent manner (Fig. 3b and Figure S2b). Moreover, dysfunction of CFTR led to disruption of tubular epithelial barrier integrity as evidenced by decreased transepithelial resistance (TER) (Fig. 3c) and enhanced migratory capability in wound healing assay (Fig. 3d). To further demonstrate the causative effect of CFTR downregulation on EMT induction, we knocked down CFTR in MDCK cells and found that CFTR silencing significantly decreased the expression levels of epithelial junction complex proteins, E-cadherin and ZO-1, whereas increased the mesenchymal markers, N-cadherin and Vimentin (Fig. 3e and Figure S2c). Similarly, knockdown of CFTR dramatically changed the expression of EMT markers including α-SMA and promoted cell migration in HK-2 cells (Figure S2d–g). In addition, fibrosis-focused PCR array illustrated that CFTR knockdown in HK-2 increased the expression of a variety of genes involved in EMT and matrix remodeling, such as COL1A2, COL3A1, MMPs, TIMP3 (Figure S3). These data indicate that suppression of CFTR function or expression is sufficient to trigger EMT process in renal tubular epithelial cells.

### CFTR negatively regulates canonical Wnt/β-catenin signaling pathway

**Figure 4:**
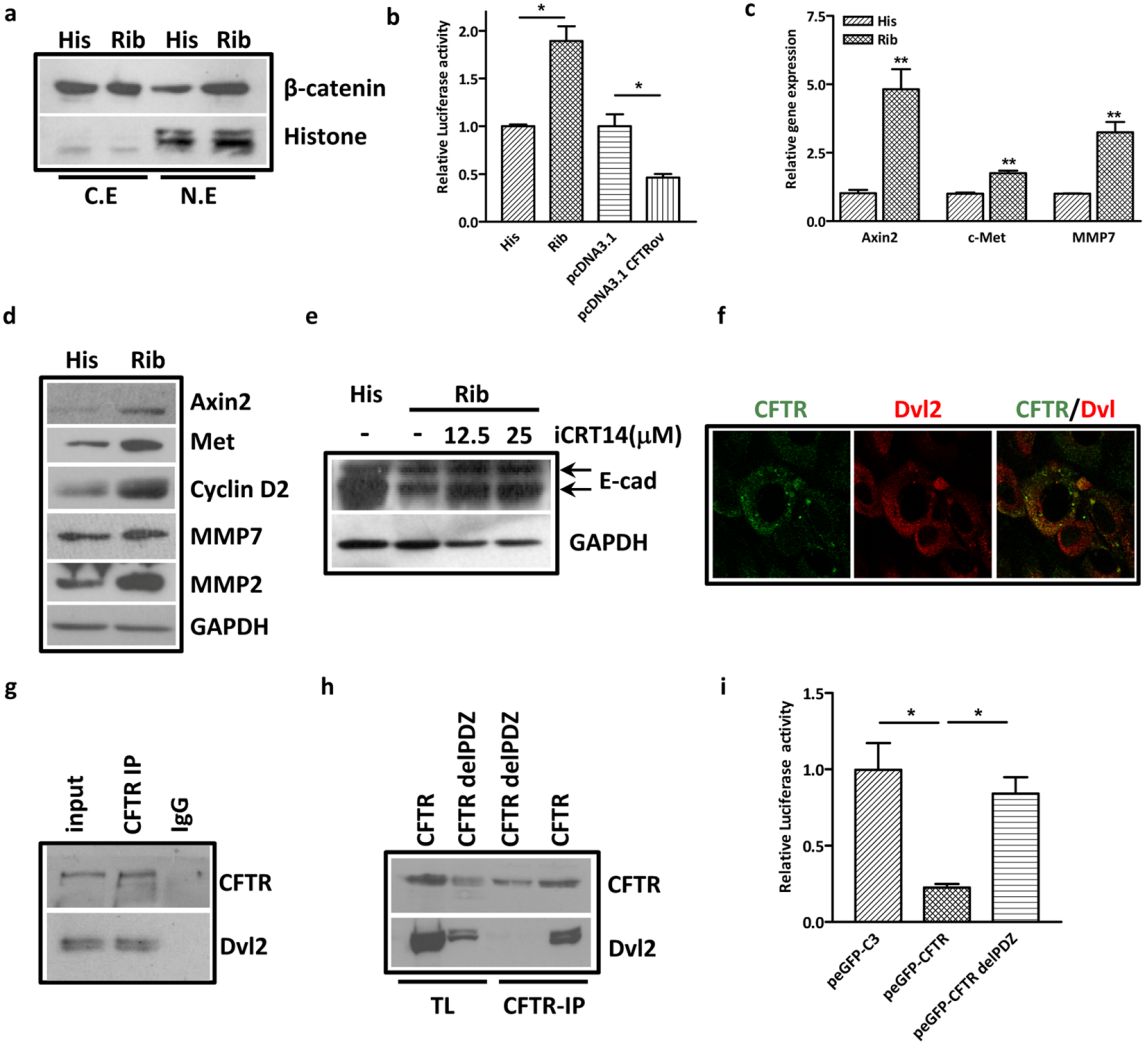
Suppression of CFTR activates β-catenin signaling pathway by interacting with DVL-2 in renal epithelial cells. (**a**) Western blot showing the increased nuclear accumulation of β-catenin in ribozymes-mediated CFTR knockdown HK-2 cells (Rib) comparing to control cells (His) transfected with control vectors (pEF6/V5-His); (N.E-nuclear extraction; C.E-cytoplasmic extraction. (Full-length blot is shown in Supplementary Figure S7g.) (**b**) luciferase activity assay showing knockdown of CFTR increases β-catenin transcriptional activity whereas overexpression of CFTR (pcDNA3.1 CFTRov) decreases β-catenin activity in HK-2 cells. Cells were lysed at 48 hours after transfected with TOPflash reporter gene (0.5 μg/ml) and Renilla-Luc (0.05 μg/ml) and the relative luciferase activity was defined as the ratio of readout for firefly luciferase to that for renilla luciferase with that of control group set as 1.0. *p < 0.05; (**c**) Real time-PCR showing increased mRNA expression of β-catenin target genes in CFTR knockdown HK-2 cells, **p < 0.01; (**d**) Western analysis showing increased protein expression of β-catenin target genes in the CFTR knockdown HK-2 cells. CFTR knockdown HK-2 cells (Rib) comparing to control cells (His) transfected with control vectors (pEF6/V5-His); (Full-length blot is shown in Supplementary Figure S7h.) (**e**) Western blot analysis shows that iCRT14 reverses decreased expression of E-cadherin in a dose-dependent manner in CFTR knockdown HK-2 cells; (Full-length blot is shown in Supplementary Figure S7i.) (**f**) Immunofluorescent staining showing the co-localization of CFTR and Dvl2 in HK-2 cells; (**g**) Co-immunoprecipitation showing the interaction of CFTR and Dvl2 in HK2 cells; (Full-length blot is shown in Supplementary Figure S7j.) (**h**) CO-IP of exogenous CFTR and Dvl2 in HEK293 cells showing lack of interaction when CFTR PDZ-binding domain is deleted; (TL: total cell lysates; full-length blot is shown in Supplementary Figure S7k.) **(i)** luciferase activity assay shows that overexpression of CFTR wild type (peGFP-CFTR), but not CFTR with deleted PDZ-binding domain (peGFP-CFTR delPDZ), increases β-catenin transcriptional activity in HK-2 cells. Cells were lysed at 48 hours after transfected with TOPflash reporter gene (0.5 μg/ml) and Renilla-Luc (0.05 μg/ml) and the relative luciferase activity was defined as the ratio of readout for firefly luciferase to that for renilla luciferase with that of control group set as 1.0. *p < 0.05.

How does CFTR affect EMT and kidney fibrosis? Since over-activation of Wnt/β-catenin signaling has been implicated in the development of kidney fibrosis^15, 17–19^ and loss of CFTR has been shown to be associated with the activation of Wnt/β-catenin in intestinal epithelium and cancer^35, 36^, we hypothesized that CFTR might interfere with Wnt/β-catenin activation in kidney epithelial cells. Indeed, we found that suppression of CFTR function or expression increased the nuclear enrichment of β-catenin protein in HK-2 and MDCK cells (Fig. 4a and Figure S4a). Moreover, using TOPflash luciferase reporter assay, we illustrated that knockdown of CFTR increased β-catenin transcriptional activity whereas overexpression of CFTR decreased it (Fig. 4b and Figure S4b). Enhanced activation of β-catenin pathway was further validated by the observed increase in the expression levels of β-catenin target genes, which have been found transcriptionally regulated by β-catenin, such as axis inhibition protein 2 (AXIN2), tyrosine-protein kinase Met (Met), matrix metalloproteinases 7 (MMP7), MMP2 and G1/S-specific cyclin-D2 (cyclin D2) (Fig. 4c,d), but not the β-catenin destruction complex (Figure S4c) in CFTR-silenced HK-2 cells. Notably, the expression of MMP2 and MMP7, which are involved in EMT process and kidney fibrosis^47^, was significantly upregulated by CFTR knockdown. We next asked whether CFTR downregulation-induced over-activation of β-catenin pathway was responsible for the observed EMT process. We first treated HK-2 cells with GSK3 inhibitor, CHIR99021 to activate β-catenin, and determined the expression of EMT markers. The results showed that activation of β-catenin significantly downregulated epithelial markers E-cadherin and ZO-1, whereas increased the expression of α-SMA (Figure S4d). Next, we asked whether the effects observed with CFTR knockdown could be reversed by inhibiting β-catenin activity. To test this, we treated CFTR knockdown HK-2 cells with a β-catenin-responsive transcription inhibitor, iCTR14 and the results showed that it reversed the CFTR knockdown-induced decrease of E-cadherin (Fig. 4e), suggesting the involvement of β-catenin signaling in the EMT process induced by CFTR suppression.

### CFTR regulates β-catenin signaling via its interaction with Dvl2

How does CFTR regulate β-catenin signaling? The cytoplasmic protein Dvl, which functions as an adaptor protein of the Wnt/β-catenin pathway, has a PDZ domain^17, 21^. Of interest, CFTR contains a PDZ-binding domain at its carboxy terminus, which has been shown to interact with the PDZ domain of a large variety of proteins^48^. Thus, we hypothesized that CFTR might interact with Dvl through the PDZ domain, thereby interfering with Wnt/β-catenin activation. We first checked whether CFTR and Dvl2, the most prominent isoform of Dvls expressed in mammals, might interact with each other in HK-2 cells. Our results showed that CFTR co-localized with Dvl2 at the cytoplasm (Fig. 4f) and physically interacted with Dvl2 as demonstrated by co-immunoprecipitation (co-IP) assay (Fig. 4g). To determine whether the interaction of CFTR and Dvl2 is dependent on the PDZ-binding domain of CFTR, which consists of four amino acids DTRL at the C-terminus, plasmids expressing full length CFTR (WT) or CFTR (ΔPDZ) (full length CFTR depleting DTRL) were transfected into HEK-293 cells, and the interaction between WT CFTR or CFTRΔPDZ with Dvl2 was determined by co-IP. Our results showed that the interaction between CFTR and Dvl2 could only be detected in WT CFTR- but not CFTRΔPDZ transfected-cells (Fig. 4h), suggesting that the physical interaction between CFTR and Dvl2 is via PDZ-binding motif. We further determined whether deletion of PDZ binding motif would affect the β-catenin transcriptional activity by using TOPflash luciferase reporter assay. Consistent with our co-IP assay, transfection of full length CFTR but not ΔPDZ dramatically suppressed β-catenin activity in HK-2 cells (Fig. 4i and Figure S4e). Taken together, these results suggest that by interacting with Dvl2, CFTR negatively regulates canonical Wnt/β-catenin signaling pathway in tubular epithelial cells.

### Disruption of CFTR exacerbates fibrotic phenotype in UUO model

**Figure 5:**
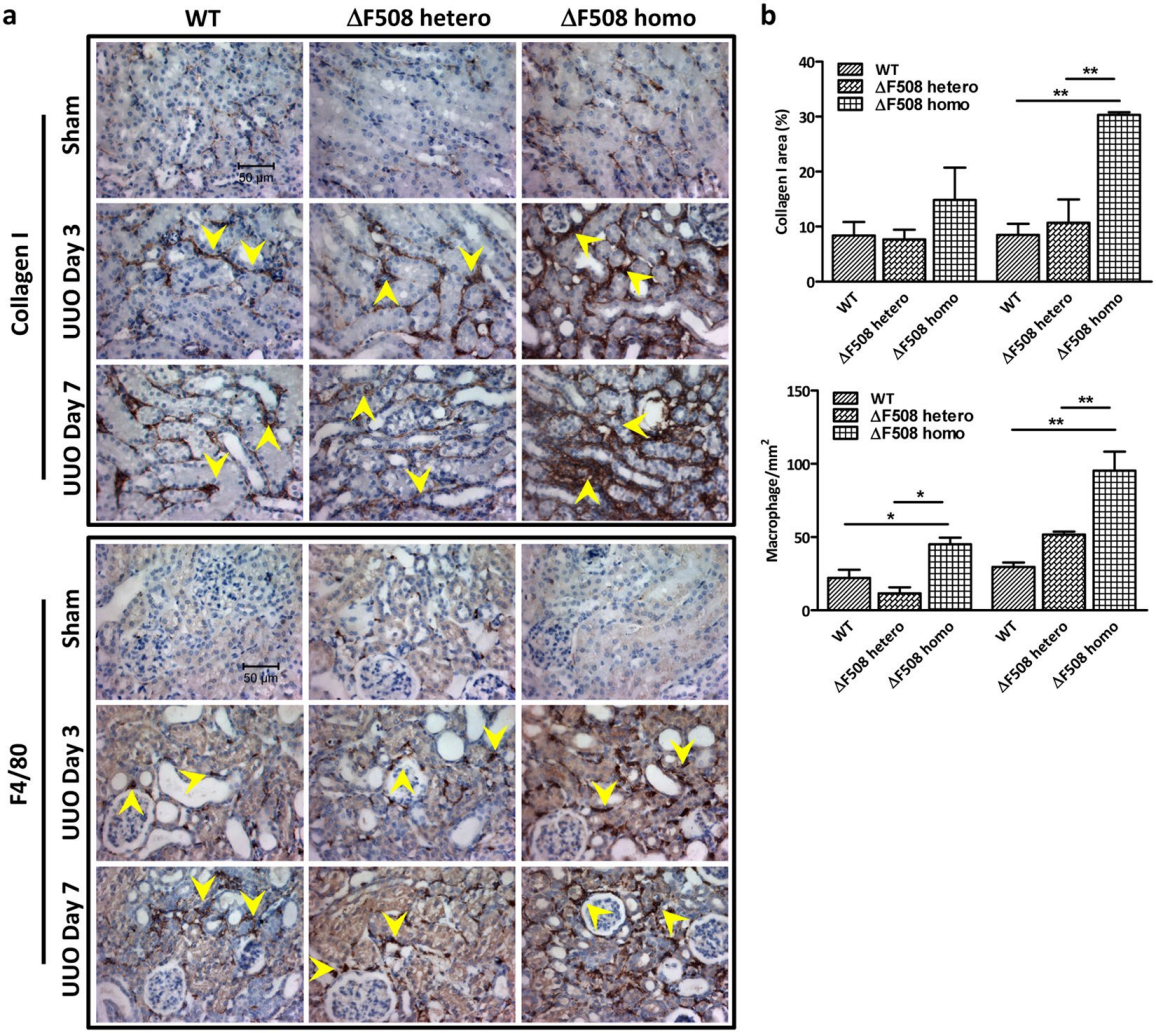
Development of kidney fibrosis is aggravated in *delta*F508 mice. (**a**) CFTR mutation aggravates kidney fibrosis manifestation in *delta*F508 mice compared to wild type mice after UUO procedure. Collagen I staining and F4/80 staining; (**b**) Quantification analysis of Collagen I staining and F4/80 staining, representing mean ± SEM from three mice. *P < 0.05, **p < 0.01, ***p < 0.001 compared with wild type mice.

**Figure 6:**
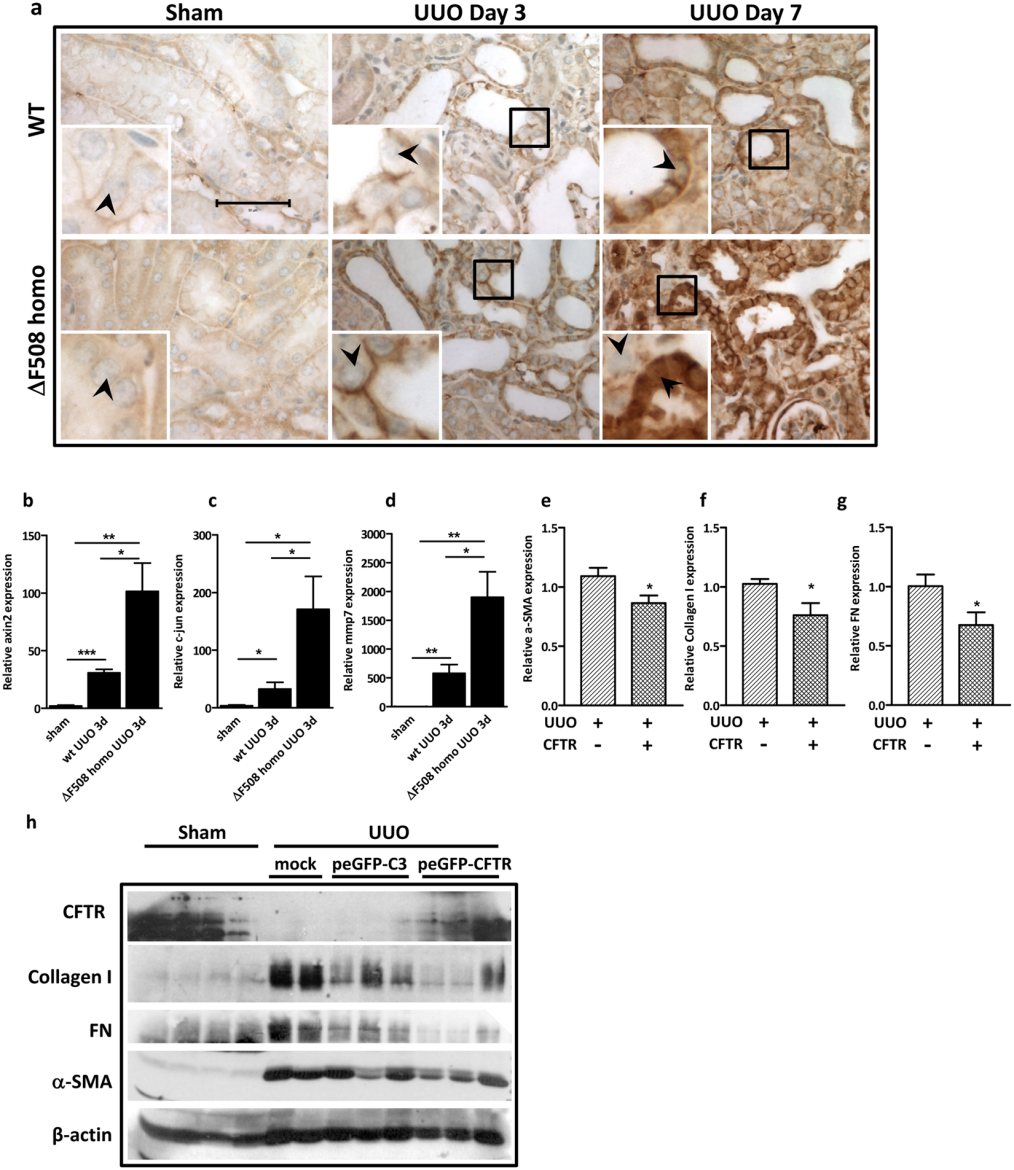
Overexpression of CFTR alleviates fibrotic phenotype in UUO model. (**a**) Immunohistochemical staining showing the dramatically increased expression of β-catenin in *delta*F508 mice compared to wild type mice after UUO. The black box marks the area enlarged, and the black arrows indicate the nuclear localization of β-catenin, scale bar = 50 µm; (**b–d**) Real time-PCR analysis showing higher expression levels of Axin-2, c-jun and Mmp7 in the *delta*F508 mice than that in the wild type mice after UUO. *p < 0.05, **p < 0.01, ***p < 0.001, n = 3; (**e–h**) CFTR overexpression abrogates kidney fibrosis in mice. (**e–g**) Real time-PCR analysis showing the decreased expression of α-SMA, collagen I and FN in peGFP-CFTR overexpression kidneys. *p < 0.05, quantification analysis represents data from 9 control and 10 CFTR overexpressed UUO kidneys. (**h**) Representative western blot showing the decreased expression of matrix protein and α-SMA in CFTR overexpressed UUO kidneys (peGFP-CFTR) comparing to control UUO kidneys (peGFP-C3) and mock UUO kidney (injected same volume of microbubbles without any DNA). (Full-length blot is shown in Supplementary Figure S7l.)

Next, we investigated whether CFTR defect could influence the development of kidney fibrosis *in vivo*. We examined the progression of obstructive renal fibrogenesis in *delta*F508 CFTR mutant mice, a well-established animal model with a mutation found in majority of CF patients^49^. The results showed that after UUO, homozygous (*delta*F508 homo) mice, developed more intensive tubule-interstitial damage including tubular atrophy, inflammatory cell invasion and interstitial extracellular matrix accumulation as demonstrated by Masson trichrome staining, collagen I deposition and macrophage infiltration in the fibrotic area of tubule-interstitium (Fig. 5a,b, Figure S5). These findings indicate that the dysfunction of CFTR leads to enhanced tubulointerstitial injury and fibrosis *in vivo*. Consistent with the *in vitro* data, the obstructive injury led to a dramatic accumulation of β-catenin in the *delta*F508 mice compared to the wild type mice (Fig. 6a). Moreover, higher expression levels of Wnt target genes, such as Axin2, c-jun and Mmp7, were observed in the UUO-treated *delta*F508 mice than that in the UUO-treated wild type mice (Fig. 6b–d), indicating aberrant activation of the canonical Wnt signaling pathway in CFTR mutant mice.

### Overexpression of CFTR alleviates fibrotic phenotype in UUO model

We next tested whether forced introduction of CFTR could alleviate the progression of renal fibrogenesis by using ultrasound-microbubble technique^50^ to deliver CFTR DNA to UUO kidneys. CFTR plasmids were delivered to the mouse kidney before the initiation of UUO procedure, and the effects of CFTR overexpression was examined 7 days after UUO treatment. Our results showed that the expression of interstitial matrix related genes such as FN, Col1a1 and α-SMA was significantly decreased at both mRNA and protein levels in CFTR-overexpressing obstructive kidneys compared to that in the vector control group after UUO (Fig. 6e–h and Figure S6a), indicating that overexpression of CFTR significantly retarded the fibrotic process. In addition, forced introduction of CFTR significantly reduced the expression of β-catenin target gene, Axin2 in UUO kidney (Figure S6b). Collectively, these results suggest that disruption of the CFTR gene exacerbates fibrotic phenotype whereas overexpression of CFTR alleviates it in the UUO model.

## Discussion

The present results have revealed a previously unrecognized role of CFTR in the development of EMT and kidney fibrosis via its regulation on β-catenin signaling pathway. In the study, we have found that suppression of CFTR downregulates the expression of Occludin and ZO-1, which are tight junction proteins, in tubular epithelial cells. This finding provides a plausible mechanism for the long-standing observation that CF patients have excessive leakage of low molecular weight (LMW) protein in the urine^51^. In addition, the demonstrated aggravation of UUO-induced EMT program and renal fibrogenesis in CFTR mutant mice may explain the observation that chronic kidney diseases are commonly seen in CF patients^29^.

Over-activation of β-catenin pathway has been shown to be associated with the development of kidney fibrosis and chronic kidney diseases^15, 52, 53^. Originally, it was reported that most of the 19 Wnt proteins and 10 Fzd receptors were increased in renal tubular cells in the UUO model of kidney fibrosis^15^. Subsequent mechanistic studies further indicated that Wnt and β-catenin were not only regulated in chronic kidney diseases, but also contributed to fibrosis development^15^. For instance, genetic overexpression of active β-catenin in tubular cells induced fibrosis feature, including epithelial dedifferentiation and EMT in mice^52, 53^. Most importantly, it has been shown recently that suppression of Wnt/β-catenin signaling, by either targeting upstream signaling factors or downstream targets ameliorates inflammation and tubulointerstitial fibrosis in mouse models^54, 55^. In the present study, we have clearly shown that β-catenin pathway is upregulated in both CFTR- knockdown renal epithelial cells and UUO-treated kidney of *delta*F508 mice (Figs 4a–d and 6a). In addition, β-catenin activator promoted EMT process, whereas β-catenin inhibitor reversed the CFTR knockdown-induced decrease of E-cadherin (Figure S4d and Fig. 4e). These results clearly indicate that CFTR negatively regulates β-catenin pathway in renal epithelial cells. In strikingly contrast, our recent studies reported that CFTR positively regulated β-catenin activity in the intestinal epithelial cells and embryonic stem cells^35, 56^. In both studies, CFTR was shown to physically interact with β-catenin, and defect of CFTR led to decreased β-catenin-mediated signaling. Of note, in this study, we did not find interaction between CFTR and β-catenin in kidney epithelial cells and kidney tissues. Instead, we found that CFTR interacted with Dvl, a key adaptor in Wnt/β-catenin signaling, and that this interaction appeared to be important for suppressing the Wnt/β-catenin signaling. In canonical Wnt signaling, Dvl functions as a scaffold protein bridging Wnt receptors and downstream signaling components via its PDZ domain^57^. In the present study, apart from Dvl2, we did not find interaction of CFTR with other β-catenin signaling components, such as Axin2 and β-catenin, in HK-2 cells (data not shown). While we cannot entirely rule out other possibilities, these results suggest that the interaction between CFTR and Dvl via PDZ domain is likely to contribute to the effect of CFTR on β-catenin activity in kidney epithelial cells. As a result, disruption of the interaction due to loss of CFTR results in aberrant activation of the Wnt/β-catenin-dependent EMT, leading to renal fibrosis. Of note, CFTR has also been found to negatively regulate the expression and activation of β-catenin in colon crypt cells and lung epithelial cells^36, 58^. Taken together, it appears that CFTR may exert differential effect on β-catenin signaling in different cellular contexts, probably via interacting with different components of the Wnt/β-catenin pathway.

Decreases in tissue oxygen levels are commonly associated with injury and repair^44^. As CFTR is expressed in a large variety of tissues, the presently observed hypoxia-induced CFTR downregulation provides a missing link between micro-environmental signaling i.e. hypoxia, and intracellular signaling pathways, i.e. Wnt/β-catenin, leading to EMT process during physiological and/or pathological conditions, such as fibrosis and cancer. On the other hand, it is very likely that dysfunction of CFTR itself augments the pathological process of kidney fibrosis in response to certain hostile environment, such as hypoxia and high glucose in CF patients. It should be noted that previous studies have established a link between TGF-β pathway and β-catenin pathway in the context of renal diseases^54, 59^. For instance, Takenaka *et al*., showed that klotho protein suppressed epithelial-mesenchymal transition in Adriamycin-induced nephropathy by inhibiting TGF-β and Wnt signaling^59^. However, in our study, we clearly showed that TGF-β, one of the key mediators of tissue fibrosis did not affect CFTR expression. Moreover, UUO-induced downregulation of CFTR was also observed in Smad3 KO mice, excluding the involvement of TGF-β signaling since Smad3 is a key player of the TGF-β signaling^60^. These results indicate that the TGF-β pathway is dissociated with the CFTR-mediated regulatory pathway in the development of hypoxia-induced renal fibrosis. Indeed, while TGF-β_1_ inhibitors have been used in the clinic to treat chronic kidney diseases; the therapeutic effect is not significant^61–63^. This observation indicates other potential players might be involved. The presently observed downregulation of CFTR expression in human fibrotic kidneys and the suppression of kidney fibrosis progression in UUO mice upon forced CFTR overexpression indicate the possibility of using CFTR as a new or combined treatment target along with TGF-β in the fibrotic kidney diseases, which warrants future investigations.

## Electronic supplementary material

Supplementary materials
