# Supplementary material for: Defective CFTR leads to aberrant β-catenin activation and kidney fibrosis
Source: Sci Rep. 2017 Jul 12;7:5233. doi: 10.1038/s41598-017-05435-5 (PMC5507915; doi:10.1038/s41598-017-05435-5)
Supplement: Supplementary file 1 — Supplementary materials [file 41598_2017_5435_MOESM1_ESM.pdf]

Defective CFTR leads to aberrant β-catenin activation and kidney fibrosis

Jie Ting Zhang[1,3*], Yan Wang[1,3*], Jun Jiang Chen[1], Xiao Hu Zhang[1], Jian Da Dong[1,5], Lai Ling Tsang[1], Xiao Ru Huang[2], Zhiming Cai[4], Hui Yao Lan[2], Xiao Hua Jiang[1,3#], Hsiao Chang Chan[1,3 #]

[1]Epithelial Cell Biology Research Center, Key Laboratory for Regenerative Medicine of Ministry of Education of China, School of Biomedical Sciences, [2]Li Ka Shing Institute of Health Sciences, Faculty of Medicine, The Chinese University of Hong Kong, Hong Kong SAR, China.

[3]School of Biomedical Sciences Core Laboratory, Shenzhen Research Institute, The Chinese University of Hong Kong, Shenzhen 518057, China.

[4]Shenzhen Second People's Hospital, The First Affiliated Hospital of Shenzhen University, Shenzhen, China.

[5] Key Laboratory of Reproduction and Genetic of Ningxia Hui Autonomous Region, Key Laboratory of Fertility Preservation and Maintenance of Ningxia Medical University and Ministry of Education of China, Yinchuan, People's Republic of China

Supplementary information:

Supplementary Figure legends

Supplementary Figure 1. Suppression of CFTR during EMT is not related to TGF-β/Smads signaling. (a) Immunofluorescent staining shows that CFTR protein is expressed at cell-cell adhesion and cytoplasm in tubular epithelium in normal human kidney, whereas significantly decreased in fibrotic kidney tissue (n=2), scale bar=50μm.

(b) MDCK cells were treated with 2ng/ml TGF-β_1 for 3 days. Western blot analysis shows that while TGF-β_1 significantly induces EMT markers, it does not have any effect on CFTR expression in MDCK cells (Full-length blot is shown in supplementary figure S7m); **(c)** Immunofluorescent staining shows that the expression of CFTR is decreased, whereas the expression of HIF-1α is significantly induced in UUO kidney in both wild type and Smad3 knockout (*smad3 KO*) mice; **(d)** Western blot analysis shows that CFTR inhibitors have no effect on the expression of phosphorylated-Smad2 in MDCK cells (Full-length blot is shown in supplementary figure S7n).

Supplementary Figure 2. Dysregulation of CFTR induces EMT in kidney epithelial cells. (a) Phase-contrast photomicrographs of MDCK cells treated with 10μM CFTRinh-172 (inh172) or 5μM GlyH101 for 48 hours; **(b)** Western blot analysis showing increased Vimentin expression and decreased Occludin expression in MDCK cells treated with different concentration of GlyH101 for 48hours; quantification analysis is shown in the lower panel, $^*p<0.05$, $^{**}p<0.01$. (Full-length blot is shown in supplementary figure S7o.); **(c)** Quantification of western blot showing significantly reduced CFTR expression in MDCK cells 48 hours post transfection with CFTR miRNA, $^*p<0.05$; **(d)** Real time-PCR showing significantly reduced CFTR expression by ribozyme (rib) –mediated CFTR knockdown in HK-2 cells; **(e)** Western blot showing decreased E-cadherin expression and increased SMA expression in CFTR knockdown HK2 cells compared to control cells; quantification analysis is shown in the lower panel, $^*p<0.05$. (Full-length blot is shown in supplementary figure S7p); **(f)** MTS assay shows no significant changes in cell proliferation in CFTR knockdown HK-2 cells compared to control cells; **(g)** Wound-healing migration assay showing enhanced cell migration in CFTR knockdown HK-2 cells compared to control cells.

Supplementary Figure 3. Focused PCR analysis showing upregulated mRNA expression of various EMT and migration-associated genes in CFTR knockdown HK-2 cells. A human fibrosis-focused PCR array was used to analyze the expression of a panel of genes involved in fibrosis. Expression levels of 19 genes show more than 2-folds change in CFTR knockdown HK-2 cells compared to control cells. ($p<0.05$)

Supplementary Figure 4. CFTR regulates β-catenin signaling. (a) Immunofluorescent staining showing the nuclear translocation of β-catenin in CFTR inhibitor-treated MDCK cells; **(b)** Western blot showing overexpression of CFTR in HK-2 cells (Full-length blot is shown in supplementary figure S7q); **(c)** Western blot showing knockdown of CFTR in HK-2 cells does not affect the expression of β-catenin destruction complex proteins (Full-length blot is shown in supplementary figure S7r.); **(d)** HK-2 cells were treated with 10μM β-catenin activator, CHIR 99021, for 48 hours. Western blot showing activation of β-catenin promotes EMT (Full-length blot is shown in supplementary figure S7s); **(e)** Western blot using primary antibody against GFP, showing the expression level of wild type CFTR (CFTRwt) and CFTR with deletion of the PDZ binding domain (CFTR delPDZ) (Full-length blot is shown in supplementary figure S7t).

Supplementary Figure 5. Defective CFTR aggravates the development of renal fibrosis. (a) Western blot showing the expression levels of CFTR in WT and ΔF508 mice (Full-length blot is shown in supplementary figure S7u); **(b)** PCR result showing the genotype of WT, ΔF508 hetero and ΔF508 homo mice. **(c)** H&E staining showing more inflammatory cell invasion in *Δ*F508 CFTR mice after UUO procedure compare

to wild type mice. Note the accumulation of inflammatory cells (arrow) in the UUO kidneys at 7 days after ligation; **(c)** Masson's trichrome stain showing more interstitial collagen expression in *Δ*F508 CFTR mice after UUO procedure compared to wild type mice.

Supplementary Figure 6. Forced expression of CFTR downregulates β-catenin target in UUO model. (a) Real time-PCR analysis showing the significantly overexpression of CFTR in peGFP-CFTR overexpression kidneys compared to peGFP-C3 control kidneys. *$p<0.05$, quantification analysis represents data from 9 control and 10 CFTR overexpressed UUO kidneys; **(b)** Representative western blot showing reduced Axin2 expression in CFTR overexpressed UUO kidneys compared to control kidneys. (Full-length blot is shown in supplementary figure S7v.) Quantification data is shown in the right panel (n=6-7).

Supplementary Figure 7. Full-length gel images of western blots.

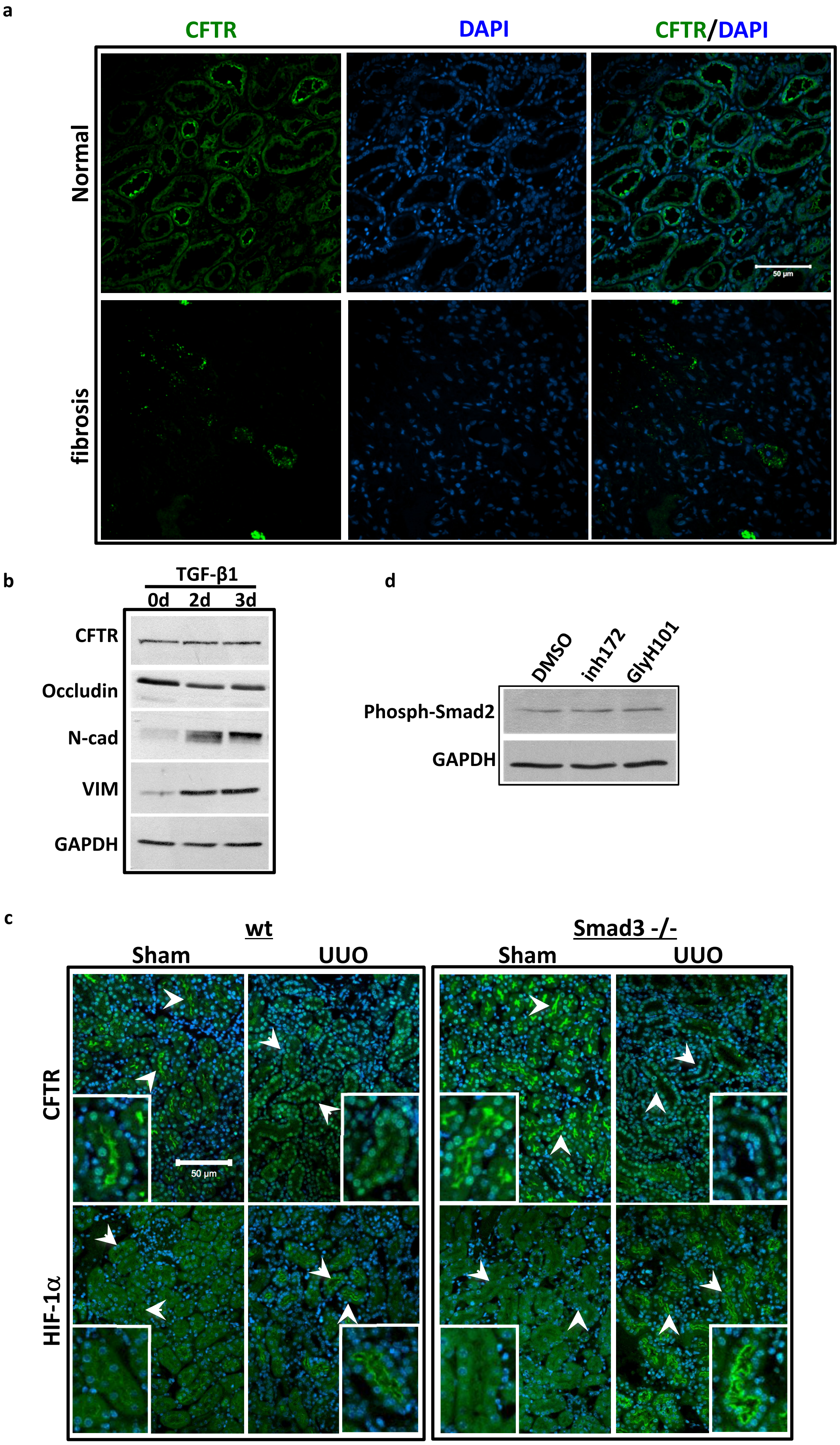
a
CFTR
DAPI
CFTR/DAPI
Normal
fibrosis
50 μm
b
TGF-β1
0d
2d
3d
CFTR
Occludin
N-cad
VIM
GAPDH
d
DMSO
inh172
GlyH101
Phosph-Smad2
GAPDH
c
wt
Smad3 -/-
Sham
UUO
Sham
UUO
CFTR
HIF-1α
50 μm

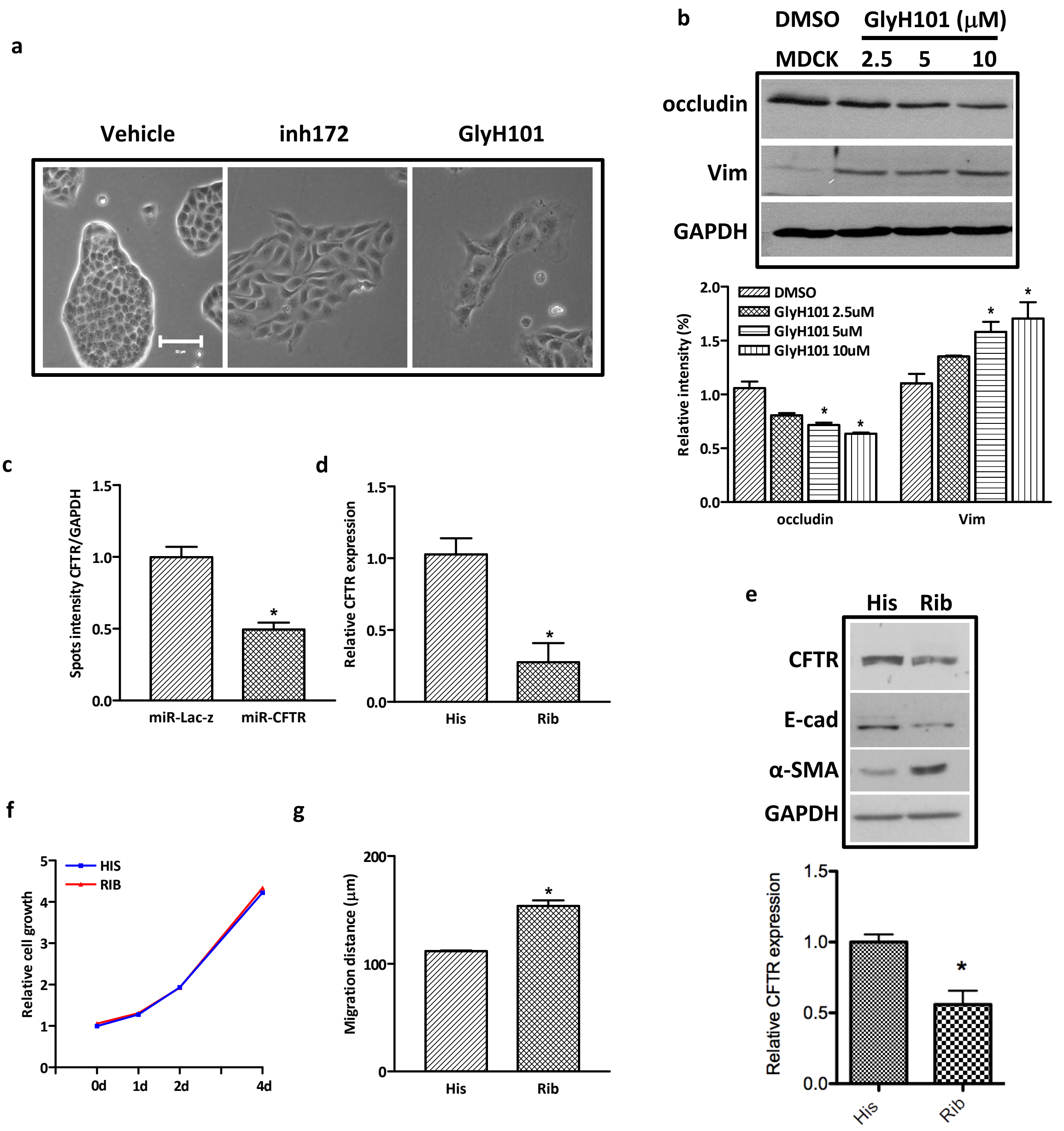
a
Vehicle
inh172
GlyH101
b
DMSO
GlyH101 (μM)
MDCK
2.5
5
10
occludin
Vim
GAPDH
DMSO
GlyH101 2.5uM
GlyH101 5uM
GlyH101 10uM
Relative intensity (%)
occludin
Vim
c
Spots intensity CFTR/GAPDH
miR-Lac-z
miR-CFTR
d
Relative CFTR expression
His
Rib
e
His
Rib
CFTR
E-cad
α-SMA
GAPDH
Relative CFTR expression
His
Rib
f
HIS
RIB
Relative cell growth
0d
1d
2d
4d
g
Migration distance (μm)
His
Rib

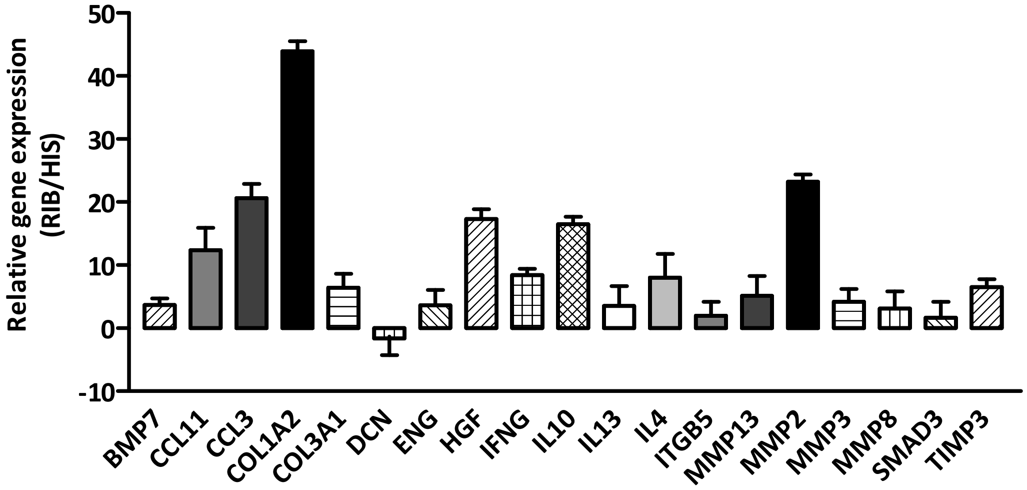
Relative gene expression (RIB/HIS)
50
40
30
20
10
0
-10
BMP7
CCL11
CCL3
COL1A2
COL3A1
DCN
ENG
HGF
IFNG
IL10
IL13
IL4
ITGB5
MMP13
MMP2
MMP3
MMP8
SMAD3
TIMP3

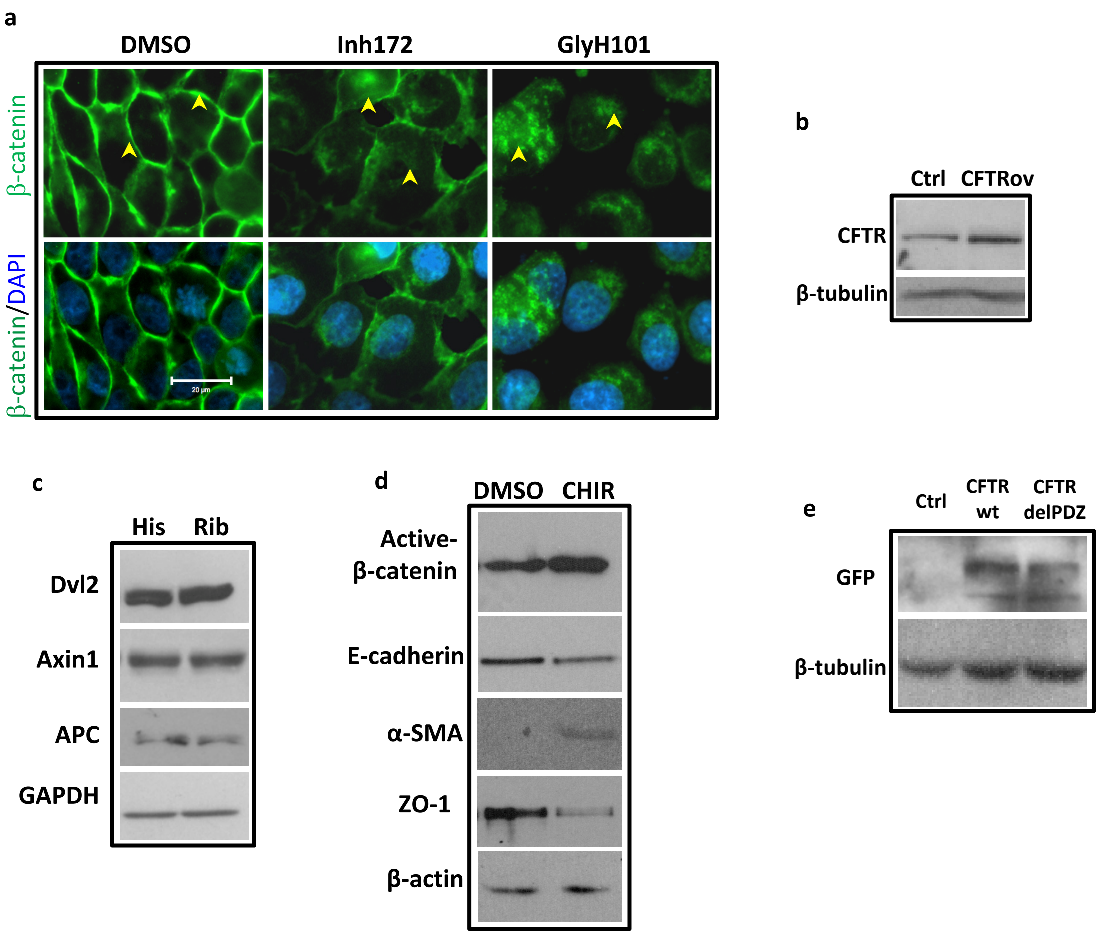
a
DMSO
Inh172
GlyH101
β-catenin
β-catenin/DAPI
b
Ctrl
CFTRov
CFTR
β-tubulin
c
His
Rib
Dvl2
Axin1
APC
GAPDH
d
DMSO
CHIR
Active-
β-catenin
E-cadherin
α-SMA
ZO-1
β-actin
e
Ctrl
CFTR
wt
CFTR
delPDZ
GFP
β-tubulin

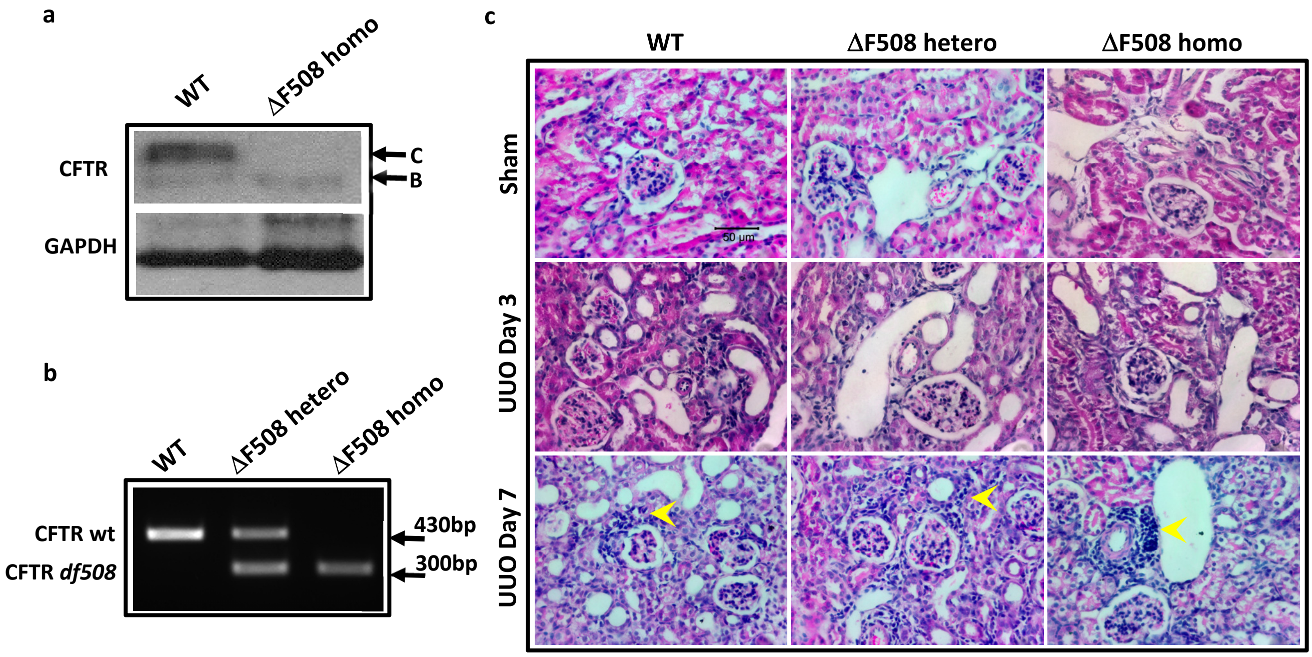
a
WT
ΔF508 homo
CFTR
GAPDH
C
B
b
WT
ΔF508 hetero
ΔF508 homo
CFTR wt
CFTR df508
430bp
300bp
c
WT
ΔF508 hetero
ΔF508 homo
Sham
UUO Day 3
UUO Day 7

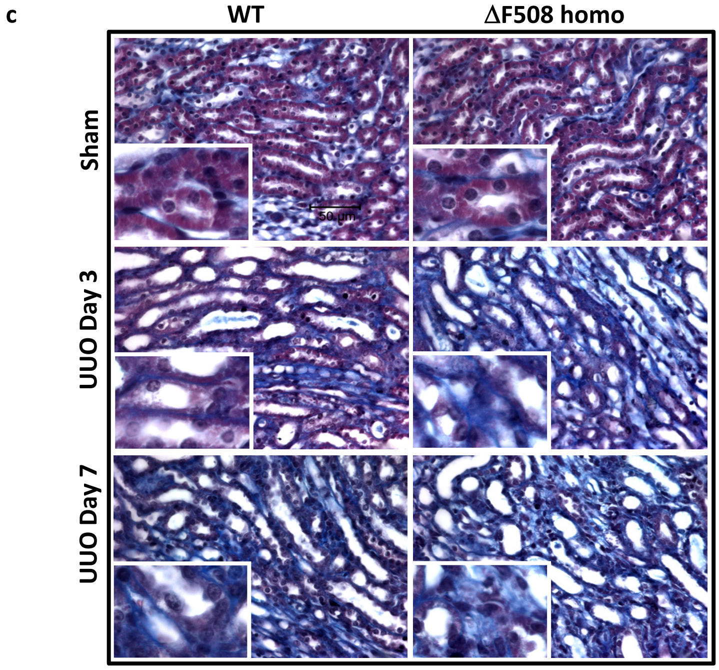
c
WT
ΔF508 homo
Sham
UUO Day 3
UUO Day 7

a

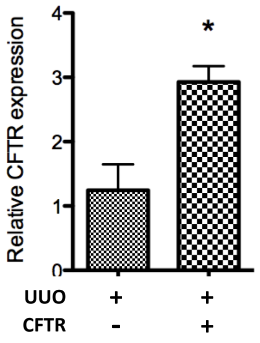
Relative CFTR expression
0
1
2
3
4
*
UUO + +
CFTR - +

b

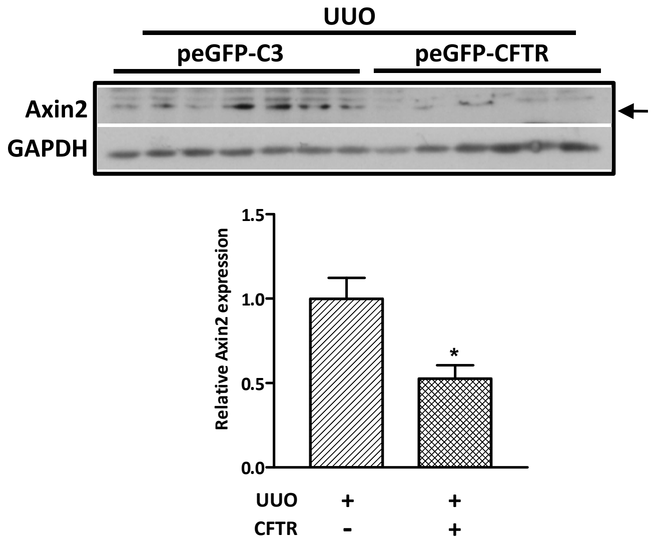
UUO
peGFP-C3
peGFP-CFTR
Axin2
GAPDH
Relative Axin2 expression
0.0
0.5
1.0
1.5
*
UUO + +
CFTR - +

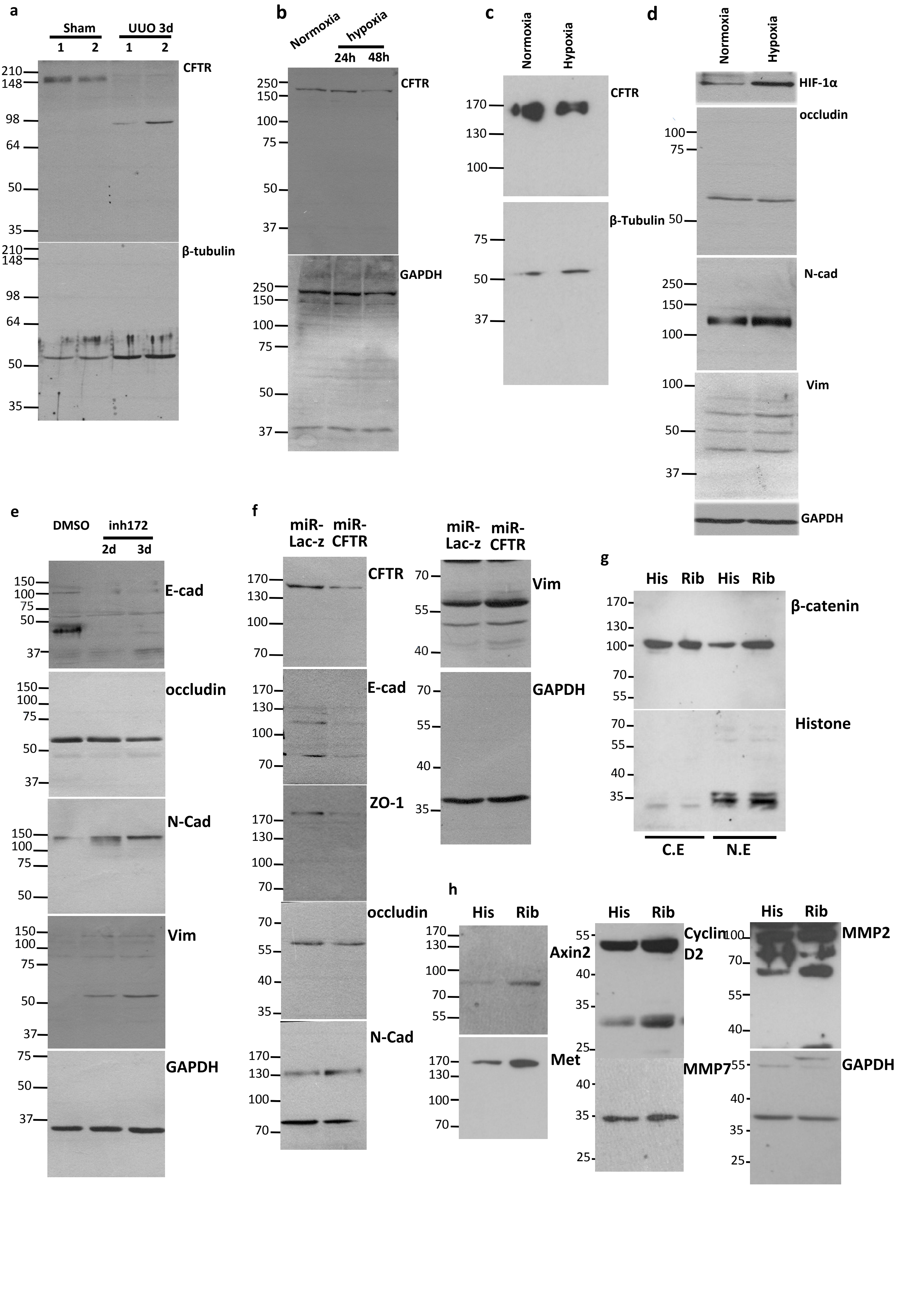
a
Sham
UUO 3d
1 2 1 2
CFTR
β-tubulin
b
Normoxia
hypoxia
24h 48h
CFTR
GAPDH
c
Normoxia
Hypoxia
CFTR
β-Tubulin
d
Normoxia
Hypoxia
HIF-1α
occludin
N-cad
Vim
GAPDH
e
DMSO
inh172
2d 3d
E-cad
occludin
N-Cad
Vim
GAPDH
f
miR-Lac-z
miR-CFTR
CFTR
E-cad
ZO-1
occludin
N-Cad
Vim
GAPDH
g
His Rib His Rib
β-catenin
Histone
C.E
N.E
h
His Rib
Axin2
Met
Cyclin D2
MMP7
MMP2
GAPDH

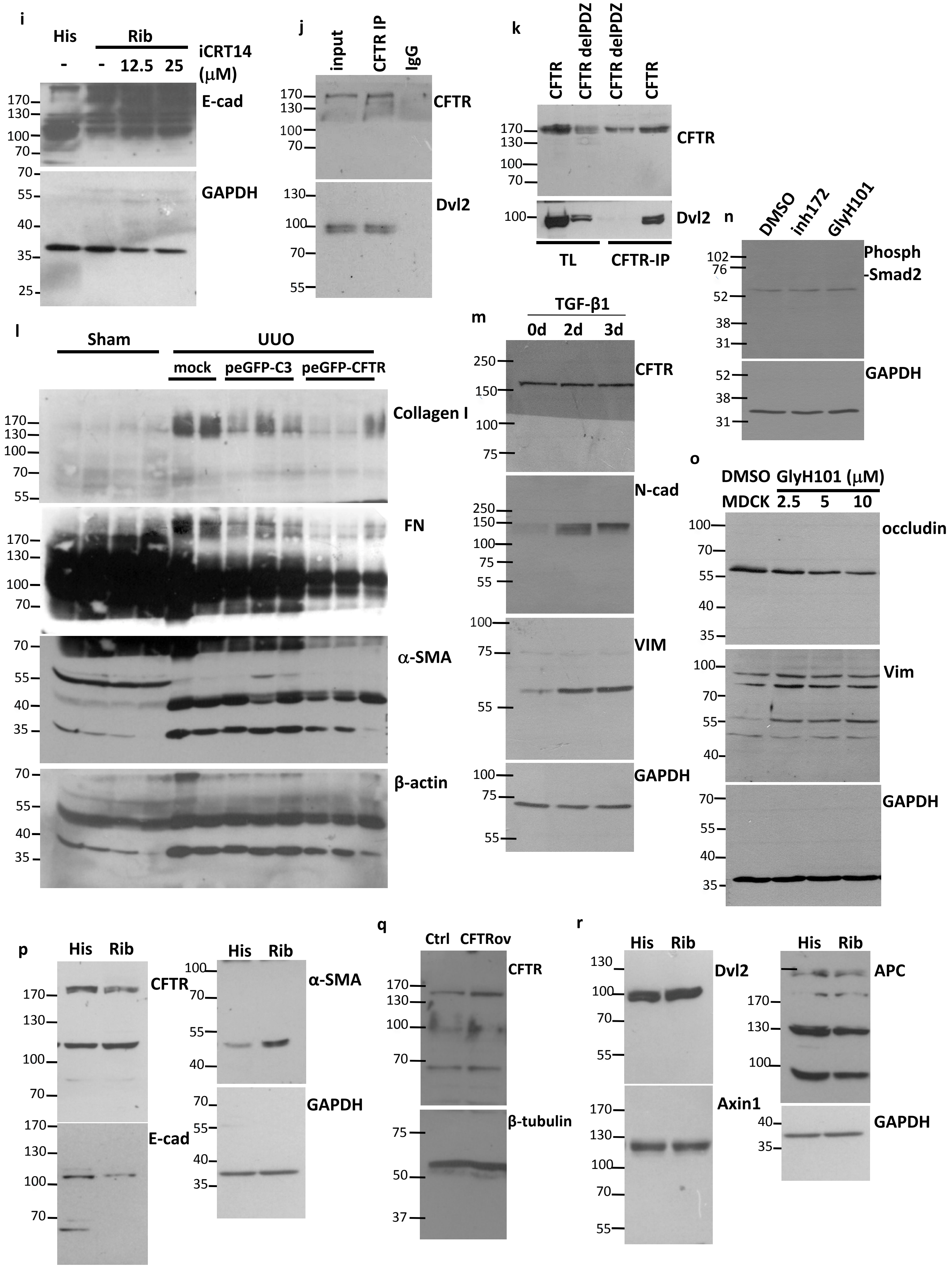
i
His
Rib
-
-
12.5
25
iCRT14
(μM)
E-cad
GAPDH
j
input
CFTR IP
IgG
CFTR
Dvl2
k
CFTR
CFTR delPDZ
CFTR delPDZ
CFTR
CFTR
Dvl2
TL
CFTR-IP
n
DMSO
inh172
GlyH101
Phosph
-Smad2
GAPDH
l
Sham
UUO
mock
peGFP-C3
peGFP-CFTR
Collagen I
FN
α-SMA
β-actin
m
TGF-β1
0d
2d
3d
CFTR
N-cad
VIM
GAPDH
o
DMSO
MDCK
GlyH101 (μM)
2.5
5
10
occludin
Vim
GAPDH
p
His
Rib
CFTR
E-cad
His
Rib
α-SMA
GAPDH
q
Ctrl
CFTRov
CFTR
β-tubulin
r
His
Rib
Dvl2
Axin1
His
Rib
APC
GAPDH

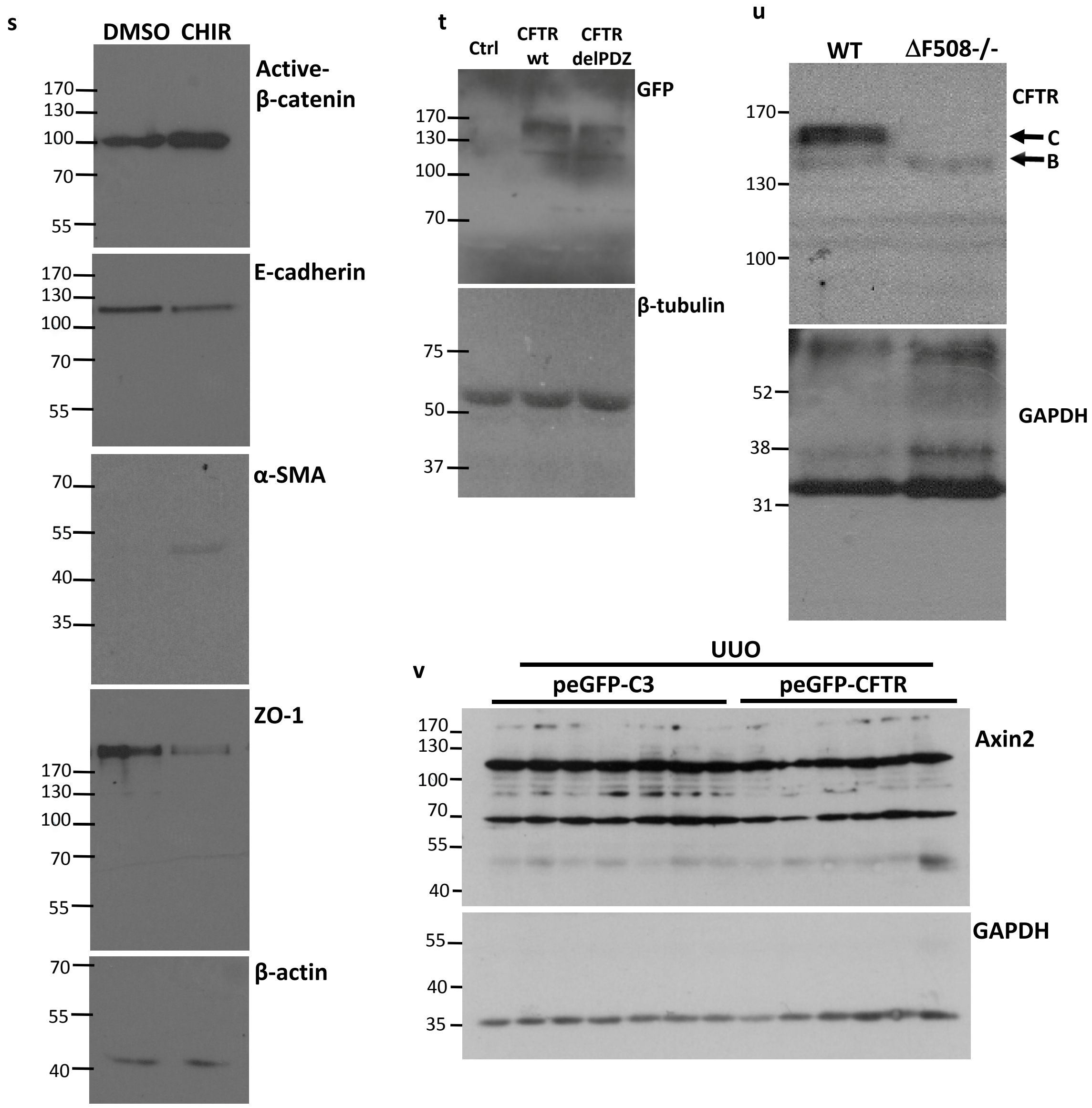
s
DMSO CHIR
Active-β-catenin
E-cadherin
α-SMA
ZO-1
β-actin
t
Ctrl
CFTR wt
CFTR delPDZ
GFP
β-tubulin
u
WT
ΔF508-/-
CFTR
C
B
GAPDH
v
UUO
peGFP-C3
peGFP-CFTR
Axin2
GAPDH

Supplementary Table1 Primer information (Human)

Gene		Primer sequence (5'-3')	size	Tm
CFTR	forward	GTGTGATTCCACCTTCTCCAA	149bp	60°C
	reverse	GCCTGGCACCATTAAAGAAA		
Axin2	forward	CTGGTGCAAAGACATAGCCA	103bp	60°C
	reverse	AGTGTGAGGTCCACGGAAAC		
C-Met	forward	TGTTCGATATTCATCACGGC	94bp	60°C
	reverse	GCATTTTTACGGACCCAATC		
MMP7	forward	GCATCTCCTTGAGTTTGGCT	103bp	60°C
	reverse	GAGCTACAGTGGGAACAGGC		
GAPDH	forward	AGGGTCATCATCTCTGCC	245bp	60°C
	reverse	CCATCACGCCACAGTTTC		

Supplementary Table2 Primer information (Mouse)

Gene		Primer sequence (5'-3')	size	Tm
Axin2	forward	ACTGACCGACGATTCCATGT	96bp	60°C
	reverse	TGCATCTCTCTCTGGAGCTG		
c-jun	forward	GGGACACAGCTTTCACCCTA	104bp	60°C
	reverse	GAAAAGTAGCCCCCAACCTC		
MMP7	forward	GCATTTCCTTGAGGTTGTCC	102bp	60°C
	reverse	CACATCAGTGGGAACAGGC		
a-SMA	forward	GGGATCCTGACGCTGAAGTA	147bp	60°C
	reverse	GTTCAGTGGTGCCTCTGTCA		
Col1A1	forward	ACATGTTCAGCTTTGTGGACC	110bp	60°C
	reverse	TAGGCCATTGTGTATGCAGC		
FN	forward	ACCTCTGCAGACCTACCCAG	120bp	60°C
	reverse	TTGGTGATGTGTGAAGGCTC		
GAPDH	forward	AACGACCCCTTCATTGAC	190bp	60°C
	reverse	TCCACGACATACTCAGCAC		
CFTR (Genotype)		TTCAAGCCCAAGCTTTCGCGAG	WT:430 bp	
		CTCCCTTCTTCTAGTCACAACCG	Hetero:430+300 bp	
		CATCTTGATAGAGCCACGGTGC	Homo:300 bp	
